# Metagenomic Sequencing Reveals that the Assembly of Functional Genes and Taxa Varied Highly and Lacked Redundancy in the Earthworm Gut Compared with Soil under Vanadium Stress

**DOI:** 10.1128/mSystems.01253-21

**Published:** 2022-01-04

**Authors:** Rong Xia, Yu Shi, Xinwei Wang, Yunling Wu, Mingming Sun, Feng Hu

**Affiliations:** a Soil Ecology Lab, College of Resources and Environmental Sciences, Nanjing Agricultural University, Nanjing, China; b State Key Laboratory of Crop Stress Adaptation and Improvement, School of Life Sciences, Henan University, Kaifeng, China; c The Key Laboratory of Plant Immunity, Jiangsu Collaborative Innovation Center for Solid Organic Waste Resource Utilization & Jiangsu Key Laboratory for Solid Organic Waste Utilization, Nanjing, China; Oregon State University

**Keywords:** community assembly, functional genes, taxa, earthworm, gut bacteria, soil bacteria, metagenomic sequencing

## Abstract

Exploring the ecological mechanism of microbial community assembly in soil and the earthworm gut in a vanadium polluted environment could help us understand the effects of vanadium stress on microbial diversity maintenance and function, as well as the mechanism of microbial mitigation of vanadium stress. Combining metagenomic sequencing and abundance distribution models, we explored the assembly of earthworm intestinal bacteria and native soil bacteria after 21 days of earthworm exposure to a gradient level of vanadate (0 to 300 mg kg^−1^) in soil. Stochastic processes dominated the assembly of both genes and taxa in earthworm gut and soil. Both the composition of taxa and functional genes in earthworm gut varied highly with the vanadium concentration, while in soil, only the taxa changed significantly, whereas the functional genes were relatively stable. The functional redundancy in soil, but not in the earthworm gut, was confirmed by a Mantel test and analysis of similarities (ANOSIM) test. In addition, vanadium detoxifying gene (VDG)-carrying taxa were more diverse but less abundant in soil than in the worm gut; and VDGs were more abundant in soil than in the worm gut. Their wider niche breadth indicated that VDG-carrying taxa were generalists in soil, in contrast to their role in the worm gut. These results suggested that earthworm intestinal and soil microbes adopted different strategies to counteract vanadium stress. The results provide new insights into the effects of soil vanadium stress on the assembly of earthworm gut and soil microbiota from both bacterial taxa and genetic function perspectives.

**IMPORTANCE** Metagenomic sequencing revealed the variation of functional genes in the microbial community in soil and earthworm gut with increasing vanadium concentrations, which provided a new insight to explore the effect of vanadium stress on microbial community assembly from the perspective of functional genes. Our results reinforced the view that functional genes and taxa do not appear to have a simple corresponding relationship. Taxa are more sensitive compared with functional genes, suggesting the existence of bacterial functional redundancy in soil, but not in the earthworm gut. These observations indicate different assembly patterns of earthworm intestinal and soil bacteria under vanadium stress. Thus, it is important and necessary to include genetic functions to comprehensively understand microbial community assembly.

## INTRODUCTION

Vanadium is a heavy metal that is distributed widely in the Earth’s crust. At low concentrations, it plays a beneficial role in nitrogen fixation and halide oxidation because vanadium is associated with the function of active sites of enzyme systems such as haloperoxidases and nitrogenase (1). In contrast, high concentrations of vanadium can cause significant toxic effects on microorganisms, plants, animals and even human health (2). Significant recent increases in worldwide vanadium production and related operational activities, i.e., vanadium mining, smelting, and further processing of vanadium products, have led to increasing amounts of vanadium pollutants entering the soil (3). Soil contains an abundance of living things, including microorganisms and invertebrates, which are sensitive to environmental stress. To survive increasing stress, i.e., accelerated vanadium exposure, invertebrates and microbial communities have evolved the capability of coexisting with heavy metals by adjusting their community composition and/or genetic functions (4). For example, heavy metal exposure (including vanadium) was reported to enrich the heavy metal-resistant bacteria in both the soil and worm gut (4, 5). Bacteria that were capable of converting highly toxic vanadium (V) to less toxic vanadium (IV), or that harbor metal effluent pump genes, were identified as vital for the vanadium reduction process, including *Bacillus*, *Clostridium*, *Comamonadaceae*, and Pseudomonas. Thus, there is the potential of using soil and earthworm intestinal microbes synergistically to remediate soil heavy metal contamination.

To determine the microbial remediation mechanism of vanadium-contaminated soil and to manipulate microbial remediation, it is important to understand the microbial diversity distribution pattern (6). Under different environmental conditions, microbial communities tended to show varying diversity patterns (7). Microbial diversity consists of alpha diversity and beta diversity, which indicate the diversity within a community and among communities, respectively. Usually, alpha diversity is characterized by richness, Shannon, Simpson, and Chao1 indices; and beta diversity consists of two components: turnover and nestedness. Turnover refers to the beta diversity caused by species replacement; when turnover dominates, disparate communities will harbor unique members. Nestedness refers to the loss or gain of species, i.e., the richness difference between samples; when nestedness dominates, communities in the sites with fewer species are the subset of the communities found at richer sites. Nestedness can be calculated based on the partitioning of beta diversity into turnover and nestedness, as described by Carvalho et al. (8). Furthermore, it can also be determined by calculating the average proportion of members from less diverse communities that occur in more diverse communities using the nestedness metric based on overlap and decreasing fill (NODF). This metric measures the average percentage of shared contacts between pairs of rows (or columns) that present a decreasing degree of ordering (9). Although microbial diversity has been investigated in various ecosystems, few studies considered the specific variance in turnover and nestedness in pollutant (i.e., vanadium)-stressed environments.

It is accepted that variance in microbial diversity is driven by their assembly process. As a long-standing central theme in microbial ecology, microbial assembly broadly refers to the mechanism that determines microbial diversity, distribution, succession, and biogeographic patterns (10, 11). Overall, microbial community assembly can be classified comprehensively into either deterministic or stochastic processes, depending on the factors predominantly driving the assembly process (12). Deterministic factors mainly consist of species characteristics, synergistic or antagonistic interactions between species, and abiotic filtering of environmental conditions (13, 14). Stochastic processes include unpredictable or probabilistic birth-death events and disturbances, such as dispersal, ecological drift, and diversification (15, 16).

As previously reported, the assembly process of a microbial community under stress is dominated by deterministic processes (12), indicating directional selection of the pollutants on the microbial communities. For example, the abundant microbial community assembly was a deterministic process that was primarily affected by petroleum content in activated sludge (17) and long-term oil-contaminated soils (18). Meanwhile, the diversity of bacteria and the stochastic ratio in community assembly gradually decreased with increasing pollution levels of livestock wastewater in pond water and sediment (19). However, the effect of heavy metal contamination on microbial community assembly remains largely unknown. In addition, most studies have explored the community assembly mechanism from a taxonomic perspective (20, 21). Microbial functional genes and taxa have both been suggested to play vital roles in understanding the microbial response to environmental stress (22, 23). Therefore, more comprehensive information would be expected when considering functional genes while simultaneously exploring the assembly rules of taxa, which could help to gain a more complete understanding of the relationship between the microbial community and the inhabited environment. Although the soil and earthworm gut are communal environments for microbial communities, contrasting abiotic environmental factors between the two systems induce varying microbial compositions and functions (4, 24). A previous study found that, because of the differences in nutrient conditions and redox status (25), earthworm intestinal bacteria harbored more abundant functional genes that are actively involved in vanadium reduction compared with soil bacteria; and by contrast, soil bacteria tend to have more abundant genes that are passively resistant to vanadium damage than earthworm intestinal bacteria (26). Therefore, we hypothesized that earthworm intestinal and native soil bacteria in vanadium-contaminated soils adopt different strategies in response to vanadium stress, manifested by the different taxa and gene assembly patterns.

In this study, we set up a trial comprising earthworm cultivation in vanadium-contaminated soil. Employing metagenomic sequencing analysis, we aimed to investigate: (i) the taxonomic bacterial community structure and functional gene distribution pattern in earthworm intestines and soil under vanadium gradient stress; (ii) the community assembly mechanisms that influence taxa and gene distribution patterns; and (iii) the feedback effects of specific community assembly patterns on detoxifying genes and taxa under vanadium stress. The results of this study might increase our understanding of the assembly mechanism of the functional genes and taxa of earthworm intestinal and native soil bacteria in vanadium-contaminated soil.

## RESULTS

### Distribution patterns of bacterial taxa and functional genes in the earthworm gut and soil.

**FIG 1:**
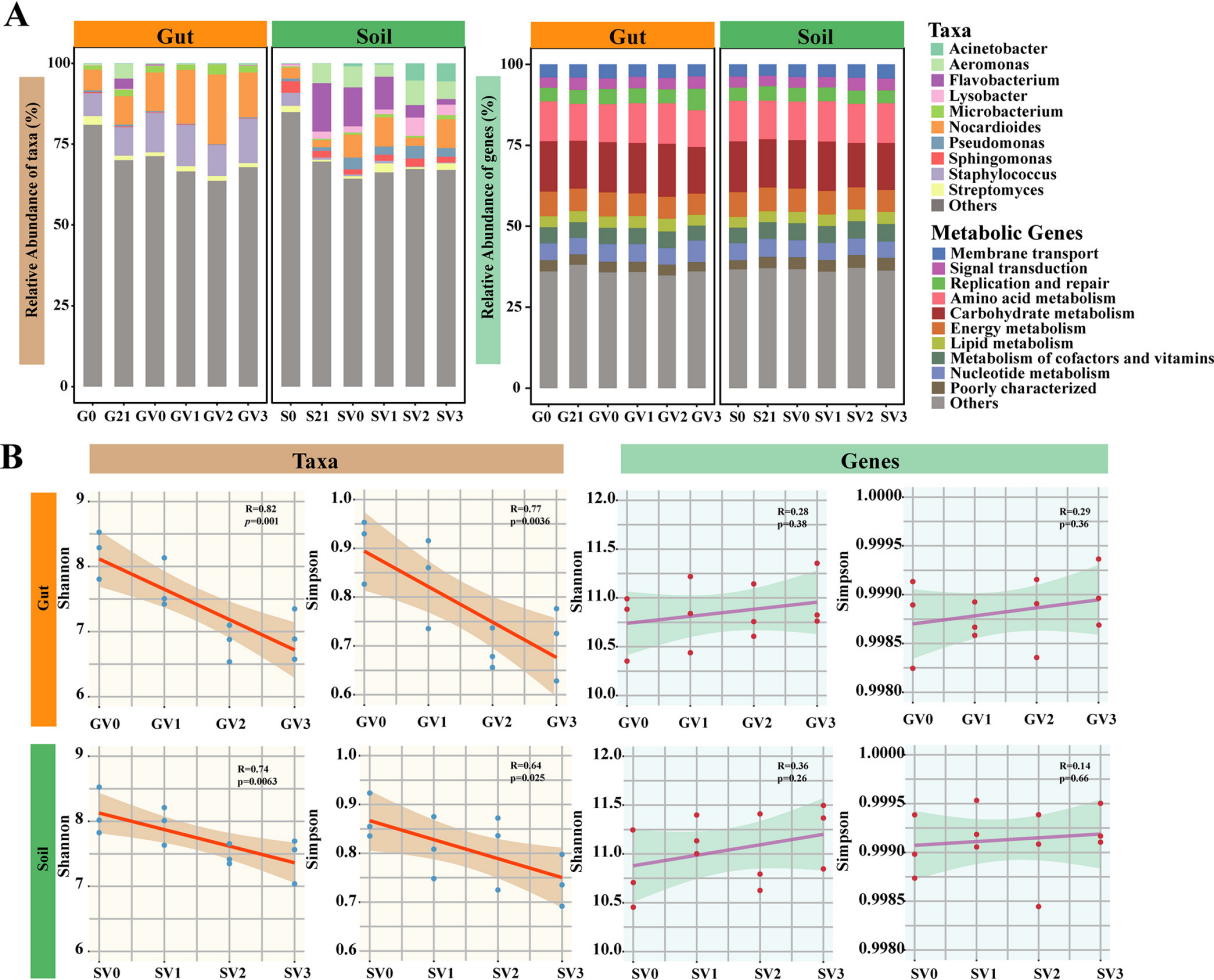
Profile of taxa and functional genes in earthworm intestines and soil across vanadium gradient concentration. (A) Taxonomic profile of the top 10 most abundant genera and functional profile of the top 10 most abundant functional genes. The rest of the taxa and functional genes are defined as “Others.” G0, the original worm gut content without microcosm trial; S0, the original soil without microcosm trial; G21, the worm gut content sampled at day 21 of the incubation; S21, the soil sampled at day 21 of the incubation; the earthworm gut contents and soils treated with 0, 100, 200, and 300 mg kg^−1^ vanadium after 21 days of incubation are represented by GV0, GV1, GV2, GV3 and SV0, SV1, SV2, SV3, respectively. (B) Correlation between alpha diversity (Shannon and Simpson index) of taxa and functional genes and vanadium concentration. The orange/green shade region represents the 95% confidence limits of the regression estimates.

The dynamics of taxonomic composition and functional structure were characterized by the relative abundance and alpha diversity of taxa and genes. We detected significant differences in the abundance of taxa at the genus level among different treatments (analysis of similarities [ANOSIM] test, *P* < 0.05), indicating that the taxonomic community composition varied distinctly under a gradient of vanadium stress (Fig. 1A). Moreover, bacterial communities in both the worm gut and soil exhibited varying dynamics under elevated vanadium stress. While the abundance of some genera, such as *Aeromicrobium*, *Clostridium*, and Klebsiella increased significantly in the earthworm intestine with increasing vanadium concentration, the abundance of other genera, including *Gemmata*, Mycobacterium, and *Marmoricola* clearly decreased (one-way analysis of variance [ANOVA] test, *P* < 0.05). In contrast, bacterial taxa that increased significantly in the soil included Acinetobacter, *Sphingobacterium*, and *Comamonas*, while *Luteitalea*, *Haloferula*, and *Shewanella* decreased (one-way ANOVA test, *P* < 0.05; Fig. S2A). As for functional genes annotated according to the Kyoto Encyclopedia of Genes and Genomes (KEGG) database, pathway level 2 remained stable across different treatments (Fig. 1A). At the level of KEGG orthologous groups (KO), the abundance of genes related to the ABC transport system in earthworm intestines increased with increasing vanadium concentrations, e.g., *ABC-2.P*, *ABC-2.A*, and *ABC.MS.S* (Fig. S2B). The abundance of functional genes in the soil was generally higher than that in the worm gut (one-way ANOVA test, *P* < 0.05; Fig. S2B). Mantel analysis indicated no clear association between the compositions of functional genes and taxa at the genus level in either the soil or earthworm gut (Mantel test, *P* > 0.05; Table S1). The alpha diversity of bacterial taxa (Shannon and Simpson diversity indices) in both the earthworm intestine and the soil decreased significantly with increasing vanadium concentration (Pearson’s correlation coefficient, two-sided tests, *P* < 0.05; Fig. 1B). In contrast, the Shannon and Simpson indices showed no clear change for functional genes in either soil or worm gut (Pearson’s correlation coefficient, two-sided tests, *P* > 0.1; Fig. 1B). The value of the alpha diversity index of bacterial functional genes in earthworm intestines and soil was higher than that for the taxa (one-way ANOVA test, *P* < 0.05).

10.1128/mSystems.01253-21.1 TABLE S1 Mantel analysis for testing the correlation between taxonomic composition and functional genes composition in earthworm gut and soil. Download Table S1, DOCX file, 0.01 MB. Copyright © 2022 Xia et al. 2022 Xia et al. https://creativecommons.org/licenses/by/4.0/ This content is distributed under the terms of the Creative Commons Attribution 4.0 International license.

10.1128/mSystems.01253-21.7 FIG S2 The abundance and diversity distribution of taxa and functional genes among different treatments. Heat map to exhibit the relative abundance of the top 50 taxa at genus level (A) and the top 50 functional genes (B). The genes are marked on the right with KO numbers. The relative abundance across treatments is indicated in color, with red representing values close to 1 and green representing values close to 0. (C) Simpson and Shannon diversity indices of species and genes in earthworm gut and soil. G0, the original worm gut content without microcosm trial; S0, the original soil without microcosm trial; G21, the work gut content sampled at day 21 of the incubation; S21, the soil sampled at day 21 of the incubation; the earthworm gut contents and soils treated with 0, 100, 200, and 300 mg kg^−1^ vanadium after 21 days of incubation are represented by GV0, GV1, GV2, GV3 and SV0, SV1, SV2, SV3, respectively. Download FIG S2, PDF file, 0.5 MB. Copyright © 2022 Xia et al. 2022 Xia et al. https://creativecommons.org/licenses/by/4.0/ This content is distributed under the terms of the Creative Commons Attribution 4.0 International license.

**FIG 2:**
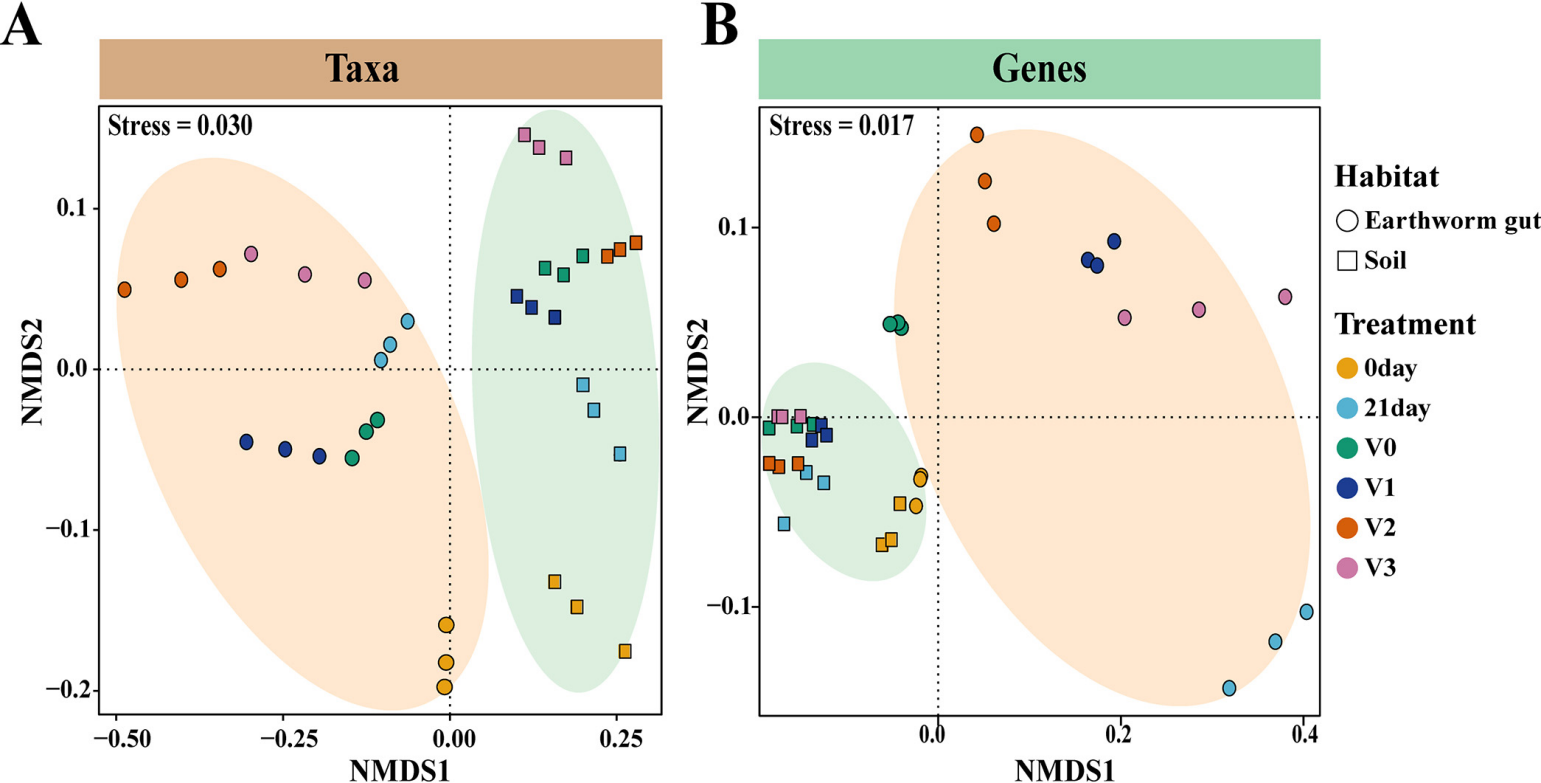
Beta diversity of taxa and genes in the earthworm gut and soil. Non-metric multidimensional scaling (NMDS) plot to display taxon (A) and functional gene (B) composition in earthworm intestines and soil under different vanadium concentrations based on the Bray-Curtis distance. The earthworm gut and soil groups are distinguished by the green and orange ellipses, respectively, based on 90% confidence according to the assumed multivariate t-distribution. G0, the original worm gut content without microcosm trial; S0, the original soil without microcosm trial; G21, the work gut content sampled at day 21 of the incubation; S21, the soil sampled at day 21 of the incubation; the earthworm gut contents and soils treated with 0, 100, 200, and 300 mg kg^−1^ vanadium after 21 days of incubation are represented by GV0, GV1, GV2, GV3 and SV0, SV1, SV2, SV3, respectively.

Beta diversity refers to the diversity among communities. We used a non-metric multi-dimensional scaling (NMDS) plot to display the Bray-Curtis distance of taxon and functional gene composition among different treatments. Samples of earthworm intestines and soils clustered into two distinct groups, indicating the difference in taxa and functional gene composition between the two habitats (Fig. 2A and B). Bacterial taxa in the worm gut and soil were clustered together according to the vanadium concentration applied, indicating that the vanadium concentration affected the taxonomic composition (Fig. 2A). In contrast, the functional genes showed different trends, separating into subgroups in earthworm intestines and clustering together in the soil at different vanadium concentrations (Fig. 2B). ANOSIM analysis indicated no clear variance in the functional gene composition in soil across gradients of vanadium stress (ANOSIM test, *P* > 0.05; Table S2), and suggested significant alterations in the functional gene composition in earthworm gut under increasing vanadium exposure (ANOSIM test, *P* < 0.05; Table S2).

10.1128/mSystems.01253-21.2 TABLE S2 ANOSIM analysis of the taxonomic/functional gene community composition between habitats (earthworm gut and soil) and vanadium concentration. Download Table S2, DOCX file, 0.02 MB. Copyright © 2022 Xia et al. 2022 Xia et al. https://creativecommons.org/licenses/by/4.0/ This content is distributed under the terms of the Creative Commons Attribution 4.0 International license.

We further investigated the turnover and nestedness of the microbial community. The results showed that no nestedness existed between taxa of the communities in the worm gut and soil, as indicted by the NODF value of taxa in communities in the two habitats being lower than that of the null model (Wald’s test, *P* < 0.05; Fig. S3A). Similarly, we detected no nestedness for the taxa in the communities along the gradient of vanadium exposure (Wald’s test, *P* < 0.05; Fig. S3B). The proportion of taxonomic beta diversity also showed that the diversity between communities tended to be dominated by turnover rather than nestedness (Fig. S3C). We also calculated the relative contribution of turnover and nestedness to beta diversity in functional genes among samples in the worm gut and soils under a vanadium gradient (Fig. S3D). Turnover was the driving factor that determined the beta diversity of the functional genes in the worm guts and soils across the treatments, except for treatments with the heaviest vanadium stress (300 mg kg^−1^), in which, conversely, nestedness became the driving factor.

10.1128/mSystems.01253-21.8 FIG S3 The nestedness of bacteria community composition in earthworm gut and soil. NODF (nestedness measure based on overlap and decreasing fills) values of taxonomic level, from phylum to genus, across vanadium gradient concentrations (A) and habitat (earthworm gut and soil) (B). Median null model NODF values are connected with dotted lines, while the NODF values calculated from the experimental data are connected with solid lines. The relative contribution of turnover and nestedness to beta diversity in taxonomic composition (C) and genetic composition (D). The pie chart shows the percentages of nestedness and turnover among different concentrations, while the length of line segment represents the percentages of nestedness and turnover between earthworm gut and soil. V0, V1, V2, and V3 represent 0, 100, 200, and 300 mg kg^−1^ vanadium, respectively. Download FIG S3, PDF file, 1.1 MB. Copyright © 2022 Xia et al. 2022 Xia et al. https://creativecommons.org/licenses/by/4.0/ This content is distributed under the terms of the Creative Commons Attribution 4.0 International license.

### Drivers of bacterial taxa and genetic function distribution patterns.

**FIG 3:**
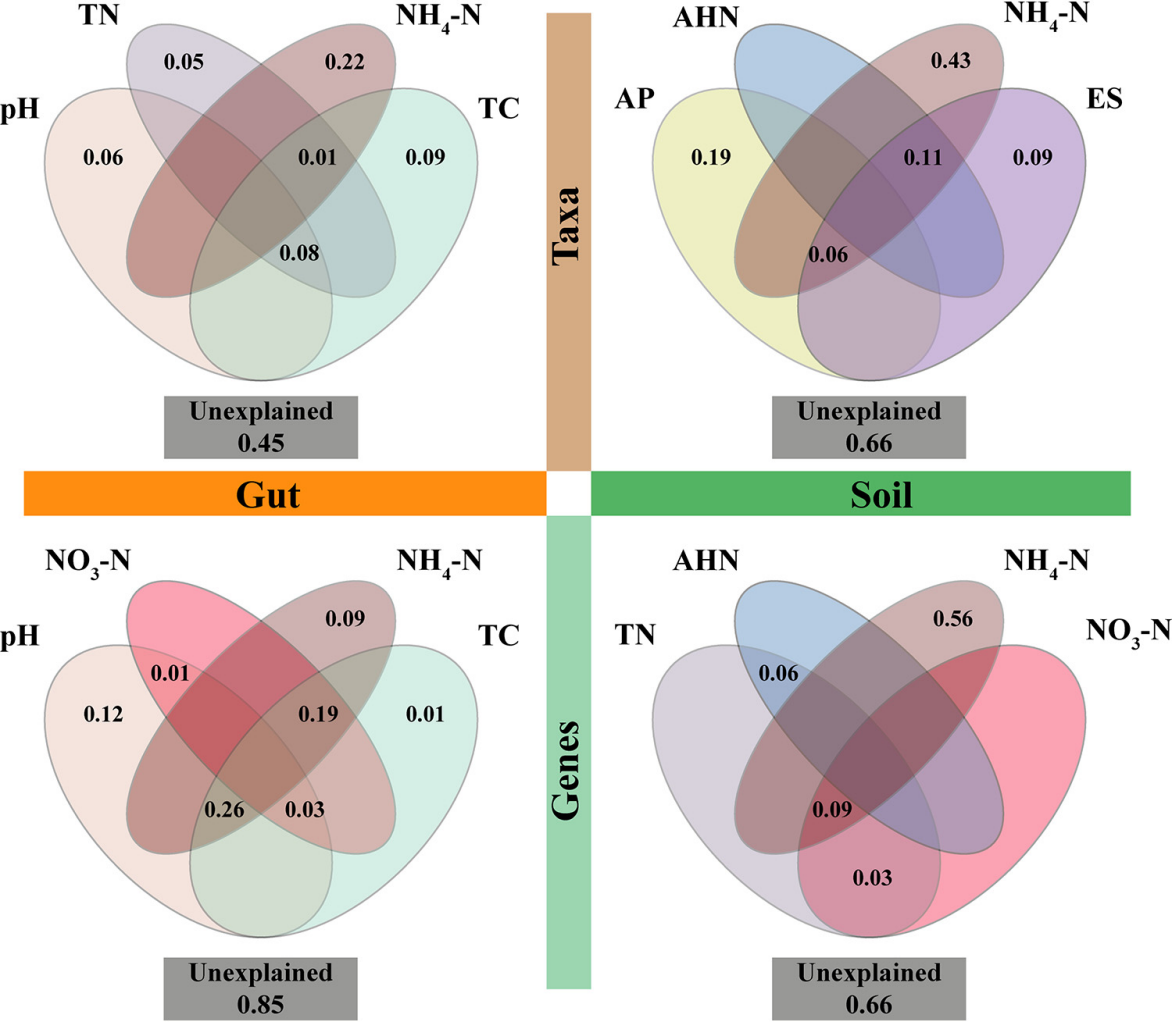
Variation partitioning analysis (VPA) to indicate the impact of abiotic factors on taxon and functional gene composition in earthworm intestines and soil. Venn diagrams show unique and joint explanatory degree of the factors to taxonomic (functional genes) composition variation. Values < 0 are not shown. TN, total nitrogen; NO_3_-N, nitrate nitrogen; NH_4_-N, ammonium nitrogen; TC, total carbon; AP, available phosphorus; AHN, alkali-hydrolysable nitrogen; AS, available sulfur.

Abiotic factors are usually important factors affecting microbial community composition. Variance partitioning analysis (VPA) was used to quantify the explanatory degree of the effects of different abiotic factors on changes in the microbial community composition and functions. Among the eight abiotic factors [pH, total nitrogen (TN), nitrate nitrogen (NO_3_-N), ammonium nitrogen (NH_4_-N), total carbon (TC), available phosphorus (AP), alkali-hydrolysable nitrogen (AHN), and available sulfur (AS)], NH_4_-N explained the greater proportion of the composition of taxa and genes in soil, and taxa in earthworm intestines, accounting for 43%, 56%, and 22%, respectively. For the functional gene composition of the earthworm gut, pH had the greatest influence (explaining approximately 12%). Forty-five percent of the taxonomic composition could not be explained in the earthworm gut and 66% in the soil. In terms of functional gene composition, 85% could not be explained in the earthworm gut and 66% in the soil (Fig. 3).

**FIG 4:**
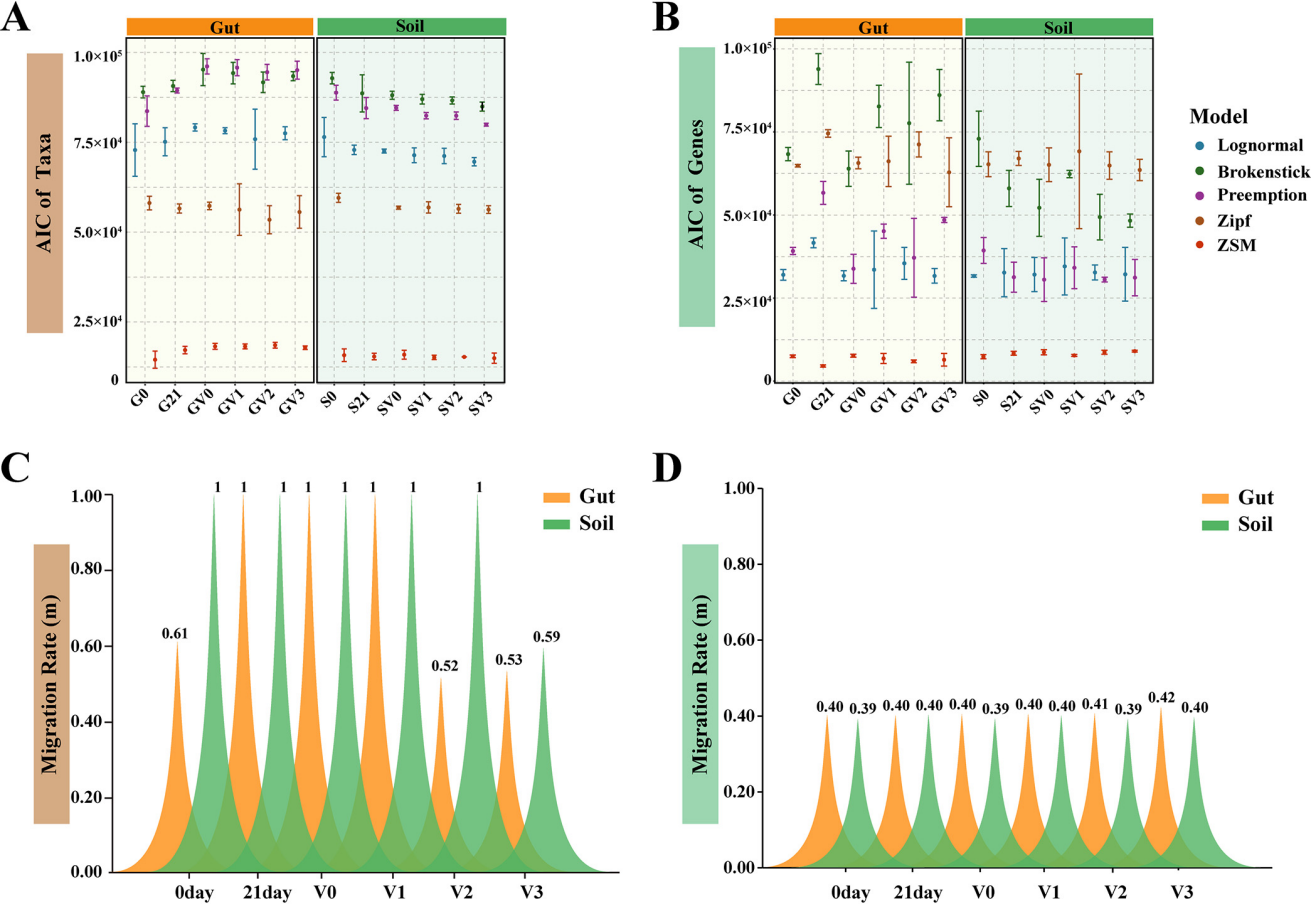
Community assembly mechanism of taxa and functional genes in earthworm intestines and soil. Akaike information criterion (AIC) values obtained from the five rank abundance distribution models to infer the dominance of niche and neutral theory in the taxa (A) and functional genes (B) assembly. The migration rate of taxa (C) and functional genes (D) across the treatments. Earthworm intestines and soil are represented in orange and green, respectively. G0, the original worm gut content without microcosm trial; S0, the original soil without microcosm trial; G21, the work gut content sampled at day 21 of the incubation; S21, the soil sampled at day 21 of the incubation; the earthworm gut contents and soils treated with 0, 100, 200, and 300 mg kg^−1^ vanadium after 21 days of incubation are represented by GV0, GV1, GV2, GV3 and SV0, SV1, SV2, SV3, respectively.

In addition to abiotic factors, it is important to understand the ecological mechanisms that drive community composition. Using LogNormal, Brokenstick, Preemption, Zipf, and zero-sum multinomial (ZSM) models (detailed descriptions are provided in Table S5), the Akaike information criterion (AIC) value was used to select the one that best fitted the rank abundance distribution of taxa and genes, therein determining whether deterministic or stochastic processes dominated the microbial community assembly (27, 28). A lower AIC value indicates a better fit of the model to the empirical data (29). We found that for bacterial taxa, the AIC value of the ZSM model in the worm gut and soil was lowest and the distribution of gene abundance was also the most consistent with the ZSM model (one-way ANOVA test, *P* < 0.05; Fig. 4A and B). This trend was consistent among the multiple models used here, suggesting that stochastic process had strong effect on the community assembly. At the same time, the bacterial taxon migration rate (*m*) in the soil decreased from 1 to 0.59 at a vanadium concentration of 300 mg kg^−1^, and declined from 1 to 0.52 at a vanadium concentration of 200 mg kg^−1^ in the worm gut (Fig. 4C). For genes, regardless of whether in the worm gut or soil, there was no significant change in the migration rate across the treatments, which were all lower than 0.5 (Fig. 4D). According to the definition of the ZSM model, which conforms to the neutral theory, a smaller migration rate represents a dispersal limitation process.

10.1128/mSystems.01253-21.5 TABLE S5 Five models used to determine the process of community assembly. Download Table S5, DOCX file, 0.02 MB. Copyright © 2022 Xia et al. 2022 Xia et al. https://creativecommons.org/licenses/by/4.0/ This content is distributed under the terms of the Creative Commons Attribution 4.0 International license.

### Vanadium detoxifying genes and taxon distribution in the earthworm gut and soil.

**FIG 5:**
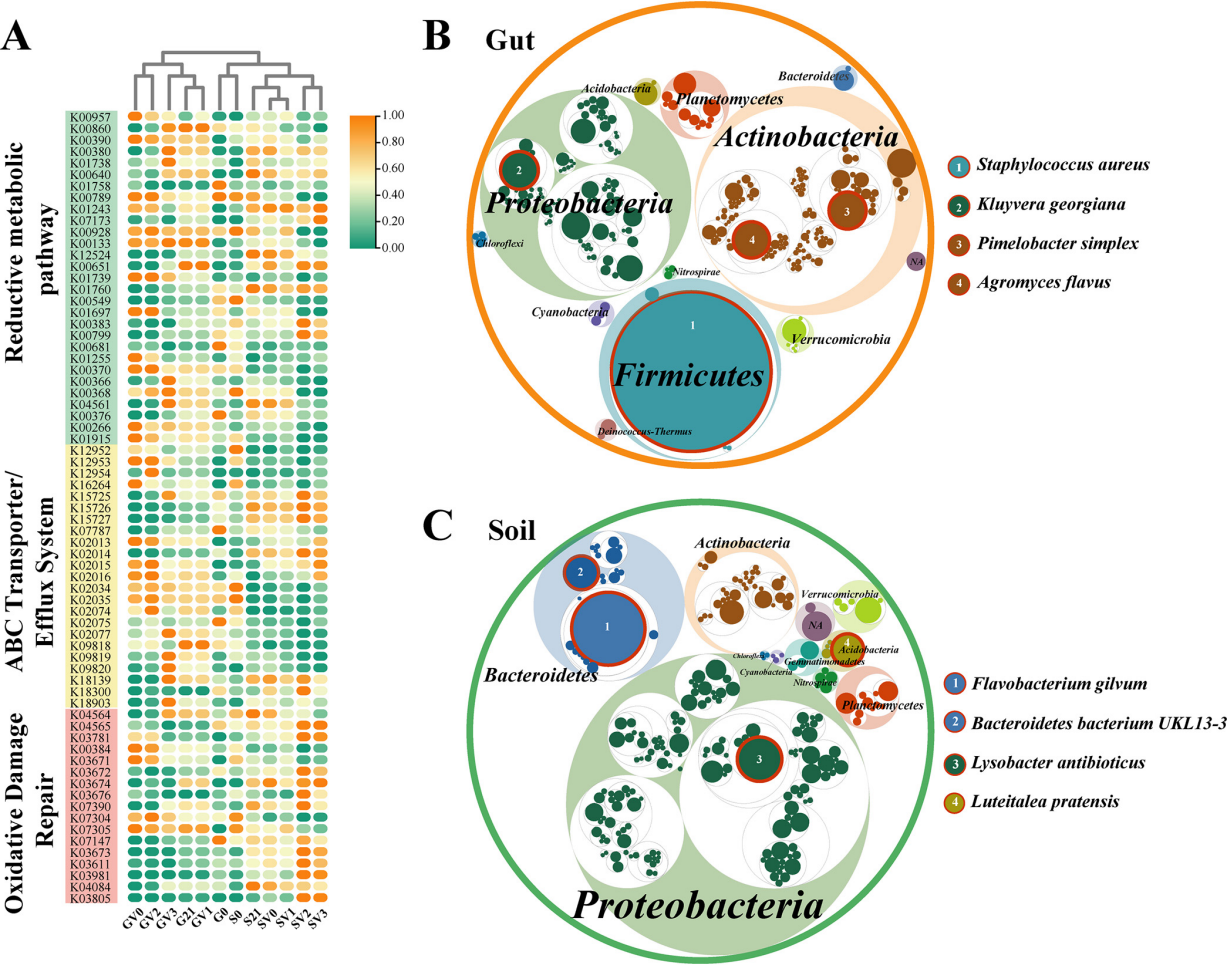
Vanadium detoxifying genes (VDGs) and VDG-carrying taxa in earthworm intestines and soils. (A) Heat maps illustrate the relative abundance distribution of VDG across different treatments. The gene names corresponding to the KO number listed on the right of the graph are showed in Table S4. The shadow color of the KO number represents the different functional gene categories (green, reductive metabolic pathway; yellow, ABC transporter/efflux system; pink, oxidative damage repair). G0, the original worm gut content without microcosm trial; S0, the original soil without microcosm trial; G21, the work gut content sampled at day 21 of the incubation; S21, the soil sampled at day 21 of the incubation; the earthworm gut contents and soils treated with 0, 100, 200 and 300 mg kg^−1^ vanadium after 21 days of incubation are represented by GV0, GV1, GV2, GV3 and SV0, SV1, SV2, SV3, respectively. Abundance distribution of VDG-carrying taxa at different taxonomic levels in earthworm gut (B) and soil (C). The largest circles represent phylum levels, and the circles with decreasing size represent classes, orders, families, genera, and species. Within the same level, the size of the circle represents the relative abundance of the taxon. The four most abundant VDG-carrying taxa at the species level are highlighted in red, and the abundance order is also numbered.

The response mechanism of earthworm intestinal and native soil microbial communities to vanadium stress was characterized by the distribution of genes related to vanadium detoxification metabolism. Functional genes encoding proteins that can actively change a metal valence state, genes encoding proteins capable of transporting heavy metals outside the cell, and those encoding proteins involved in the repair of the oxidative damage caused by heavy metals were selected from all genes obtained by metagenomic sequencing, including genes related to nitrate reduction, sulfate reduction, cysteine metabolism, and genes encoding heavy metal efflux pumps, ABC transporters, and antioxidant enzyme systems. We defined these genes as vanadium detoxifying genes (VDGs). The overall abundance of VDGs in the soil was higher than that in the worm gut (one-way ANOVA test, *P* < 0.05; Fig. 5A). Bacterial taxa carrying these VDGs in the worm gut and soil also varied greatly from one another. By screening the protein sequences encoding VDGs in bacterial sequences, we identified the VDG-carrying taxa with potential to protect bacteria from vanadium damage. VDG-carrying taxa in the worm gut were mainly distributed in *Firmicutes* (53.8%), *Proteobacteria* (17.3%), and *Actinobacteria* (17.1%) (Fig. 5B and Fig. S4A). Staphylococcus aureus in *Firmicutes*, Kluyvera georgiana in *Proteobacteria*, Pimelobacter simplex and Agromyces flavus in *Actinobacteria* were the four most abundant VDG-carrying taxa at the species level, in order (Fig. 5B). However, *Proteobacteria* and *Bacteroidetes* were the main phyla that harbored VDGs in the soil, of which the relative abundances were 47.6% and 23.7%, respectively (Fig. S4B). The most abundant VDG-carrying taxa at the species level in soil were *Flavobacterium gilvum* and *Bacteroidetes bacterium UKL 13-3* in *Bacteroidetes*, Lysobacter antibioticus in *Proteobacteria*, and Luteitalea pratensis in *Actinobacteria* (Fig. 5C).

10.1128/mSystems.01253-21.9 FIG S4 The relative abundance of VDG-carrying taxa in the earthworm gut (A) and soil (B), The pie chart represents the abundance distribution at the phylum level. The top 10 genera of the three most abundant phyla are shown in the bar chart. Download FIG S4, PDF file, 1 MB. Copyright © 2022 Xia et al. 2022 Xia et al. https://creativecommons.org/licenses/by/4.0/ This content is distributed under the terms of the Creative Commons Attribution 4.0 International license.

10.1128/mSystems.01253-21.4 TABLE S4 The KO numbers and corresponding names of vanadium detoxifying genes (VDGs) which falls into three different categories. Download Table S4, DOCX file, 0.02 MB. Copyright © 2022 Xia et al. 2022 Xia et al. https://creativecommons.org/licenses/by/4.0/ This content is distributed under the terms of the Creative Commons Attribution 4.0 International license.

**FIG 6:**
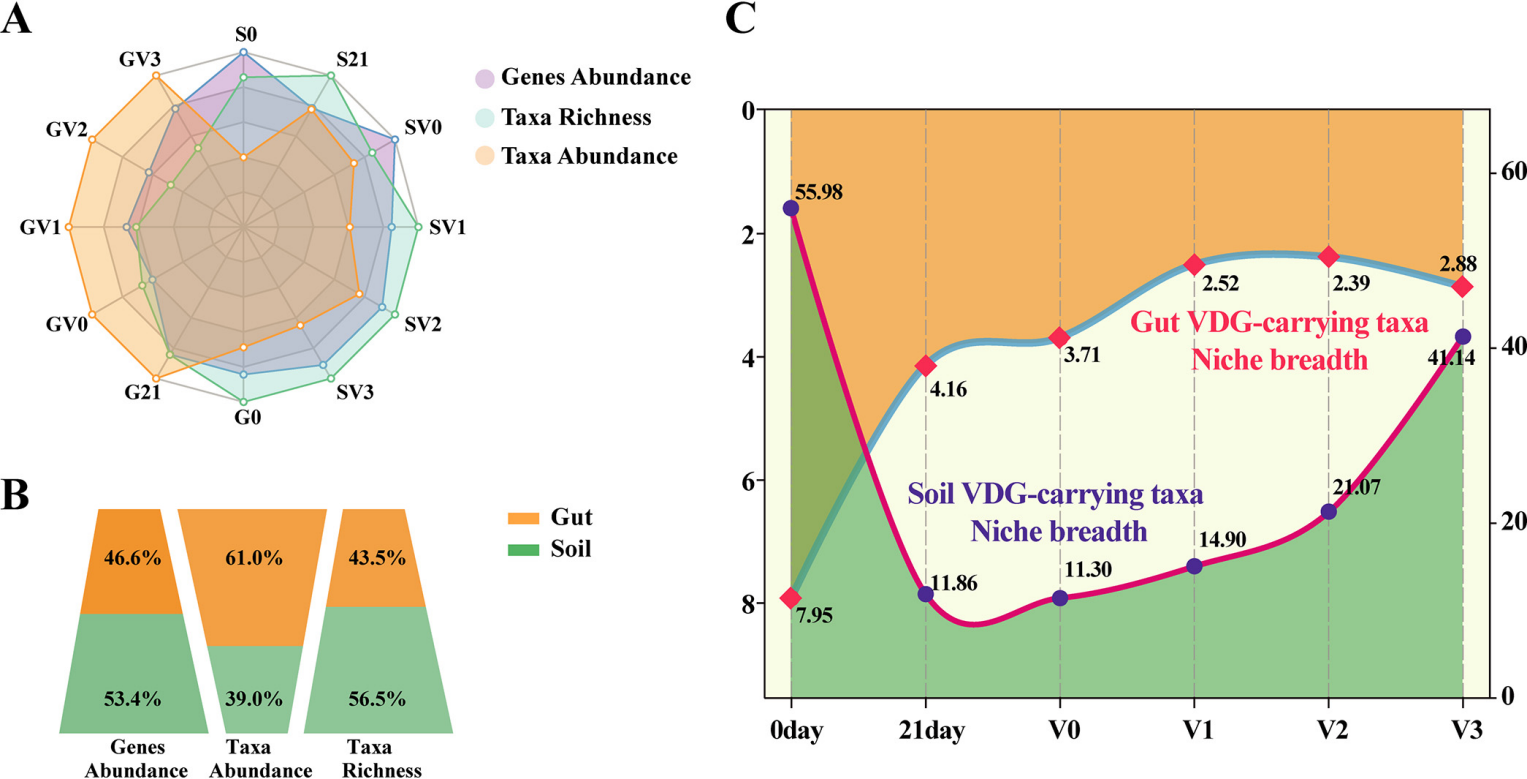
Profile of vanadium detoxifying genes (VDGs) and VDG-carrying taxa in the worm gut and the soils. (A) Radar graph to analyze the average relative abundance of VDGs and VDG-carrying taxa, and the average relative richness of VDG-carrying taxa under different treatments. (B) The ratio of VDG abundance, VDG-carrying taxonomic abundance, and richness of VDG-carrying taxa between the earthworm intestines and the soil. (C) Mean habitat niche breadths of VDG-carrying taxa in earthworm intestines (orange) and soils (green). G0, the original worm gut content without microcosm trial; S0, the original soil without microcosm trial; G21, the work gut content sampled at day 21 of the incubation; S21, the soil sampled at day 21 of the incubation; the earthworm gut contents and soils treated with 0, 100, 200, and 300 mg kg^−1^ vanadium after 21 days of incubation are represented by GV0, GV1, GV2, GV3 and SV0, SV1, SV2, SV3, respectively.

We also found that the proportion of VDG abundance to the total gene abundance in the worm gut was lower than that in the soil (one-way ANOVA test, *P* < 0.05). Considering the VDGs in the worm intestine and soil in total, the VDGs in the worm gut and soil accounted for 46.6% and 53.4%, respectively. In contrast, the relative abundance of VDG-carrying taxa in the worm gut was higher than that in the soil among different treatments (one-way ANOVA test, *P* < 0.05). Besides, VDG-carrying taxa in the worm gut and soil accounted for 61.0% and 39.0%, respectively. Regarding the richness of VDG-carrying taxa (i.e., the number of VDG-harboring taxa), we observed 43.5% of taxa in the worm gut and the remaining 56.5% in the soil (Fig. 6A and B). Consequently, in the worm gut, less abundant VDGs were distributed in fewer VDG-carrying taxon categories, resulting in the higher abundance of these taxa. In soil, more abundant VDGs presented in more VDG-carrying taxon categories, leading to the lower abundance of these species. We calculated the niche breadth of VDG-carrying taxa in the worm gut and soil, and found that the niche breadth of VDG-carrying taxa in the soil ranged from 11.3 to 56.0, which was significantly higher than that in the worm gut (2.4 to 8.0). Moreover, the niche breadth of VDG-carrying taxa in the soil increased significantly with an increase in vanadium concentration, but no similar phenomenon was observed in the worm intestines (Fig. 6C).

## DISCUSSION

### Compared with genes, taxa were more sensitive to the vanadium stress only in soil.

In the present study, we detected that the taxonomic composition varied greatly, while genetic functions showed no clear variance across different vanadium treatments (Fig. 1A). Mantel analysis confirmed that there was a decoupling relationship between taxonomic composition and functional composition (Mantel test, *P* > 0.05; Table S1). Meanwhile, although ANOSIM analysis indicated no clear variance in the functional gene composition in soil across gradients of vanadium stress (ANOSIM test, *P* > 0.05; Table S2), significant alteration of the functional gene composition in the earthworm gut was observed in response to increased vanadium exposure (ANOSIM test, *P* < 0.05; Table S2). Therefore, we only found evidence of functional redundancy in soils. Given that diversity contributes to ecosystem stability (30), the lack of functional redundancy in earthworm intestinal bacteria might reflect the lower diversity of functional genes in the earthworm gut than in soil, making its functional composition more susceptible to increased vanadium concentration than soil (Fig. S2C). Specific functions can be broadly distributed in distant microbial taxa to counteract environmental stress (31). Miao et al. (32) conducted bench experiments to assess the taxonomic and genetic functions in response to 1,4-dioxane-contaminated groundwater under different biostimulation and bioaugmentation treatments. The taxonomic composition of the microbial community changed dynamically under different treatment conditions, while the functional pathways either remained stable or eventually returned to the original state. The similar taxonomic variation and functional stability suggested the broad existence of functional redundancy in microbial communities.

Taxonomic diversity decreased (both Shannon and Simpson indices) in the worm gut and soil with an increase in vanadium concentration (Pearson’s correlation coefficient, two-sided tests, *P* < 0.05; Fig. 1B). The Shannon index had a higher correlation with vanadium concentration than Simpson index. By contrast, the functional gene diversity showed no significant change (Pearson’s correlation coefficient, two-sided tests, *P* > 0.1; Fig. 1B). Furthermore, the diversity index of the functional genes was greater than that of the taxa (Fig. 1B), suggesting that the increase in vanadium concentration led to increased stress on the microbial community and a subsequent decrease in microbial taxonomic diversity. This is consistent with Wang et al.’s study (33) on the influence of pollutants on microbial communities, which demonstrated that taxa responded variably to heavy metal stress, resulting in the presence of resistant and sensitive taxa in the polluted environment. Under severe pollution stress, sensitive taxa were more vulnerable to pollution, and only a small proportion of resistant taxa survived. Some sensitive taxa disappear under severe pollutant stress; therefore, taxonomic diversity decreases in the microbial community. In contrast, functional genes and taxa do not appear to have a simple corresponding relationship, as in previous studies, which revealed the prevalent decoupling of taxonomic composition and functional composition in microbial systems (31, 34, 35). Functional redundancy exists broadly in microbial communities, thus even different taxa may carry the same functional genes (36). Consequently, bacterial functions do not follow the evolving route of the taxa under the stress of exogenous pollutants synchronically, potentially because the factors that shape the taxonomic and functional composition are commonly different. Changes in the function of microorganisms do not depend on changes in their taxonomic composition, leading to the decoupling of bacterial taxa and functions (37). By contrast, most functions are not exclusive to a particular group of bacteria because of convergent evolution and horizontal gene transfer among microorganisms (38). The category of a taxon can determine the general function potential; however, one type of function does not represent a specific taxonomic branch (34). Consequently, unless the environmental disturbance is strong enough to cause the directed extinction of an entire taxon performing a particular function, the loss of taxa caused by minor environmental disturbances does not lead synchronically to a rapid decline in functional diversity. These observations indicate the potential resilience and buffering capacity of microbial ecosystems under heavy metal pollution.

### Turnover drove the beta diversity of taxa and functional genes in earthworm and soil.

Our results demonstrated that turnover drove the beta diversity of taxa and functional genes in earthworm and soil. As far as we know, turnover and nestedness are the two main factors that drive beta diversity. Turnover represents the replacement of taxa in communities, and disparate communities tend to harbor a distinct taxonomic structure if turnover dominates. Nestedness represents the gain or loss of taxa among disparate communities, thereby resulting in varying richness of taxa. If nestedness is dominant, a community with fewer taxa is prone to become the subset of the community with more taxa (9). The predominant role of turnover as the driving factor of the taxonomic and genetic function composition indicated that the worm gut and soil contained unique taxa and functional genes at low levels of vanadium stress (i.e., 100 and 200 mg kg^−1^). While under the heaviest vanadium stress (300 mg kg^−1^), the beta diversity of genes between earthworm gut and soil communities was dominated by nestedness (Fig. S3D). The reason might be that the earthworm gut could filter and screen complex functional genes from soil at high vanadium concentration, which led to the nestedness among communities. Many studies have shown that the phenomenon of turnover primarily shaping beta diversity is common in microbial ecosystems (39, 40), as was shown in the present study for taxonomic beta diversity. In contrast, few studies have explored the drivers of bacterial genetic function beta diversity, let alone under a gradient of pollutant stress. According to Chen et al. (41), antibiotic resistance gene (ARG) diversity could be attributed to a greater extent by turnover rather than by nestedness in lake sediments. However, our results showed that a high vanadium concentration (300 mg kg^−1^) converted the dominant factor of genetic function’s beta diversity from turnover to nestedness. In view of the mechanisms responsible for nestedness stemming from community thinning (42), we considered that a high vanadium concentration was sufficient to cause differences in the gene abundance between worm gut and soil.

### Stochastic processes dominated the bacterial community assembly in earthworm gut and soil.

The abundance distribution patterns at specific spatial and temporal scales can be used to explore potential ecological processes underlying the mechanism of community assembly, to further understand the formation and maintenance mechanism of ecosystem diversity (43). We found that the community assembly of earthworm intestinal and soil bacterial taxa was more likely to conform to the neutral theory under vanadium stress. This is in accordance with a previous study, which also found that stochastic processes influence the assembly of microbial communities under drought stress (44). This might be because the community size is smaller under the stress condition, as shown in our study as the decline in taxonomic diversity (Fig. 1B), and the individual is more prone to extinction because of accidental factors relative to the unstressed condition, thereby empowering the effect of ecological drift in the community assembly. According to the ZSM model, the taxon migration rate decreased when the concentration increased to 200 mg kg^−1^ in the worm gut and to 300 mg kg^−1^ in the soil, suggesting the important role of dispersal limitation processes on microbial assembly patterns. Moreover, given that deterministic processes also played a relative role in microbial assembly, we found that abiotic factors indeed explained the variation in taxonomic and functional gene composition (especially for taxonomic composition with high degree of explanation) (Fig. 3). This supports the theory that deterministic and stochastic processes both play important roles in microbial community assembly, and the difference lies in which process is dominant in different environments (11, 27).

The importance of function to the ecological process of microbial assembly has been recognized; therefore, function, rather than taxa, might be proposed as a more appropriate parameter to understand patterns of microbial diversity (45). As indicated by the “lottery hypothesis,” linking taxonomic assembly to functional assembly, while exploring the ecological mechanisms of microbial community construction, is important. In a “competitive lottery mode” based on this hypothesis, the ecological niche and stochastic processes affected microbial community assembly simultaneously (23). The hypothesis holds that a particular niche prepares for a group of taxa with the same nutrient metabolism or other ecological characteristics, but the available space within the niche will eventually be occupied by the taxa with suitable functions that arrived first. This means that the selection of functions is a deterministic process, and which taxon enters the ecological niche is as random as winning the lottery (46, 47) (a scheme showing the concept of “competitive lottery model” is shown in Fig. S5). In the bacterial communities associated with the green macroalga *Ulva australis* that fitted the “competitive lottery model,” it was found that the alternative microbial communities existed in ecosystems, the core functions of which are identical, while the composition at taxonomic level varies significantly (45). This mechanism was useful to explain our finding that functional redundancy existed in the soil under vanadium stress. Together, both niche theory and neutral theory play a role in the community assembly of native soil bacteria under vanadium stress. At the same time, the selection of functional composition is more deterministic, while the construction of taxa is more stochastic.

10.1128/mSystems.01253-21.10 FIG S5 Conceptual figure of “competitive lottery model.” Background colors are used to distinguish bacteria with different functions. Bacteria with the same background color are considered members of a guild of functionally equivalent species. Solid arrows indicate the deterministic selective effect of the ecological niche on species with a particular function. Dotted arrows represent random recruits into the niche from functionally equivalent species. Red stars represent species “winning” chance to enter the niche space. Download FIG S5, TIF file, 1.1 MB. Copyright © 2022 Xia et al. 2022 Xia et al. https://creativecommons.org/licenses/by/4.0/ This content is distributed under the terms of the Creative Commons Attribution 4.0 International license.

### Earthworm intestinal and native soil bacteria adopted different strategies to counteract vanadium stress.

In resource-limited environments, bacteria are believed to expand their niche breadth to ensure survival (48, 49). By contrast, in environments with abundant resources, the bacterial metabolic function can reach peak performance (50). There is a trade-off between peak performance and performance breadth; therefore, if a bacterium becomes an specialist in a certain field, the opportunity to have multiple metabolic functions will be lost, resulting in narrowing of the niche breadth (51). Luan et al. (48, 52) proved that bacterial individuals had lower maximum fitness and a weaker ability to perform biogeochemical functions in extreme environments. To obtain sufficient energy to maintain cell survival and reproduction, the niche breadth of the bacterial community increased. The nutrient concentration of the worm gut is higher than that of the soil (48). Soil bacteria are exposed first and more directly to vanadium contaminants compared with worm gut bacteria; therefore, in this study, vanadium stress might have caused a more serious disturbance to the soil habitat than the worm gut. As a result, the niche breadth of VDG-carrying taxa in the soil increased along with increasing vanadium concentration, whereas the variation in the worm gut was not significant among treatments (Fig. 6C). VDG-carrying taxa in the original soil (S0) and earthworm gut (G0) could not compete with other dominant species for resources in the community when they were not subjected to vanadium stress, thus the niche breadth of VDG-carrying taxa was relatively high. Moreover, because of relative nutrient deficiency and greater stress in soils, the relative abundance of VDG-carrying taxa in soil was lower than that in the worm gut. However, the results also showed that the niche breadth of VDG-carrying taxa in the soil was significantly higher than that in the worm gut. The wider the niche breadth, the less specialized the taxon, and the taxon would tend to be a generalist, and *vice versa* (53). Therefore, the VDG-carrying taxa in the worm gut in our study tended to be specialists, while they tended to be generalists in the soil. In addition, the abundance and diversity of VDG-carrying taxa and VDGs were different in the earthworm gut and soil bacteria (Fig. 6A and B). This might reflect the distinction in gene expression between the earthworm gut and soil and the limitations of our understanding of VDG-carrying taxa, and it is not enough to prove the decoupling between taxa and function. However, these results, together with the difference of niche breadth of VDG-carrying taxa in the earthworm gut and soil, suggest that earthworm intestinal and soil microbes adopted different strategies to counteract vanadium stress.

### Concluding remarks.

Our results show that both stochastic and deterministic processes play a role in community assembly in the soil and earthworm gut, although stochasticity is more important. Among the abiotic factors we explored, NH_4_-N is the most important factor in explaining taxonomic and gene variation in the soil and earthworm gut, which is the key factor that should be considered in regulation of microbial communities. In addition, functional redundancy existed in the soil but was absent in the earthworm gut. Compared with those in the earthworm gut, soil functional genes tended to be more redundant, manifested as insensitivity to changes in vanadium concentration. This was possibly because soil microbial communities have higher genetic diversity than that in earthworm intestines.

In this study, the abundance distribution pattern was used to explore the assembly process of the microbial community. This method might have limitations because different mechanisms might produce the same abundance pattern. Therefore, it is worth considering the exploration of the optimal analytical method for microbial community assembly in future studies. In addition, by analyzing metagenomic data, we found some VDG-carrying taxa in the *Firmicutes*, *Proteobacteria*, and *Actinobacteria*. Their actual functions need to be verified later by obtaining pure strains. This was the first study that aimed to understand the microbial assembly processes in the soil and earthworm gut under vanadium stress. The findings will provide baseline information for the regulation of microbial communities and for further exploration of vanadium detoxification bacteria in vanadium-stressed environments. Considering the limitation of using KO annotations, which likely group distant orthologs, more evidence is needed in future studies to prove the changes in microbial functions under vanadium stress, such as the combination of multiple-omics studies.

## MATERIALS AND METHODS

### Experimental procedures.

Adult *Metaphire guillelmi* earthworms were used to conduct a culture experiment using simulated vanadium-contaminated soil. The soil was collected from farmland in Nanjing, China (118°46’48” E, 32°2’24” N) (background values of vanadium: total vanadium: 65.3 ± 3.2 mg kg^−1^; pentavalent vanadium: 17.2 ± 2.2 mg kg^−1^; tetravalent vanadium: 45.2 ± 3.1 mg kg^−1^). According to preliminary results and the Chinese environmental quality standard for agricultural soil (GB15618-2009), 0, 100, 200, and 300 mg kg^−1^ of vanadium were selected as treated concentrations in the experiment by adding 0, 50, 100, and 150 mg sodium vanadate (NaVO_3_) to 500 g soil. In order to obtain artificially vanadium contaminated soil, the soil was incubated for 28 days to make the heavy metals reach a relatively stable state (54, 55). Three incubators were set for each concentration treatment. After incubation, 10 healthy earthworms of uniform length were placed in each incubator for 21 days and the soil moisture content was maintained at 15% to 20% during this period. Earthworms cultured for 21 days were anaesthetized with alcohol and intestinal contents were obtained according to the method described in our previous publication (26). Briefly, the earthworms were treated with anhydrous ethanol and rinsed with sterile water. Then the complete intestinal contents were obtained by dissecting below the worm’s clitellum region using sterile scissors and scalpel. The obtained intestinal contents were stored in a −20°C refrigerator. Intestinal contents were analyzed with soil samples for subsequent metagenomic analysis (the sample label of earthworm intestines and soil treated with 0, 100, 200, and 300 mg kg^−1^ vanadium were GV0, GV1, GV2, GV3 and SV0, SV1, SV2, SV3, respectively). At the same time, the worm gut (G0) and original soil (S0) without culture experiment was collected; and to eliminate differences in microbial communities caused by the culture process, worms were cultured in autoclave sterile soil for 21 days and the intestines were sampled and analyzed as G21, along with the cultured soil as S21. Three replicates were set for each sample. The physicochemical properties of earthworm intestines and soil are listed in Table S3.

10.1128/mSystems.01253-21.3 TABLE S3 Abiotic factors of earthworm intestinal contents and soil under different treatments. Download Table S3, DOCX file, 0.02 MB. Copyright © 2022 Xia et al. 2022 Xia et al. https://creativecommons.org/licenses/by/4.0/ This content is distributed under the terms of the Creative Commons Attribution 4.0 International license.

### DNA extraction and metagenome sequencing.

DNA was extracted from earthworm intestinal contents (0.5 g for each sample) and soils (0.5 g for each sample) using the FastDNA kit (MP Biomedicals, CA, USA) using the specific steps described in the manufacturer’s operation manual. DNA concentration and purity were determined using a NanoDrop ND1000 spectrophotometer (NanoDrop Technologies, USA) (56). Extracted DNA was sent to Shanghai Personal Biotechnology Company (Shanghai, China) for metagenomic sequencing. Based on the Illumina high-throughput sequencing platform, the whole genome shotgun (WGS) method was used to randomly break the extracted metagenomic total DNA into short fragments (57). Libraries of inserts of appropriate length were built and paired-end (PE) sequencing was performed on these.

FastQC was used for quality control of raw data. The quality distribution of metagenomic sequencing data was uniform, and the peak width was between Q36 to Q40, indicating the high quality (Fig. S1A). In addition, the distribution of guanine-cytosine (GC) content was close to normal distribution, indicating that there was no contamination in the libraries (Fig. S1B). The raw data was then screened and filtered. The Cutadapt (v1.2.1) was used to identify and remove the adapter sequence at the 3’ end (required matching length with the adapter ≥ 10 bp, and the base mismatch rate ≤ 20%). Then the sliding window approach was used to screen the sequence quality: the window size was 5 bp, and the sequence began to move from the first base position at the 5’ end. The sequence was truncated from the first window whose average mass value was lower than Q20 (the average base sequencing accuracy was ≥ 99%). The length of the preserved sequence after truncation should not be less than 50 bp, and there is no ambiguous base. BWA (http://bio-bwa.sourceforge.net/) was used to remove potential earthworm genome contamination. After above processing, the clean data set containing 1,154,011,748 reads was obtained.

10.1128/mSystems.01253-21.6 FIG S1 Quality information for metagenomic data. (A) Mean sequence quality distribution. The abscissa represents the average quality value of a single sequence, and the ordinate represents the number of sequences under the corresponding quality value. (B) Mean GC content distribution. The abscissa represents the value of GC content of a single sequence, and the ordinate represents the number of sequences. Red is the actual distribution curve of GC content for all sequences, and blue is the theoretically predicted distribution curve. The higher the coincidence degree of the two curves, the lower the bias of the sequence components, and the GC content distribution is as expected. (C) The rarefaction curve at species level. Download FIG S1, PDF file, 0.8 MB. Copyright © 2022 Xia et al. 2022 Xia et al. https://creativecommons.org/licenses/by/4.0/ This content is distributed under the terms of the Creative Commons Attribution 4.0 International license.

After preprocessing and quality control of the original sequences obtained by sequencing, megahit (https://hku-bal.github.io/megabox/) was used to perform *de novo* assembly of the paired-end sequences with the parameter setting of K-mer ∼ [27, 127] to construct Contigs and Scaffolds sequences. A total of 1,836,214 contig and 1,929,381 scaffolds larger than 1 kb were obtained. MetaGeneMark (http://exon.gatech.edu/GeneMark/metagenome) was used to conduct gene prediction on Scaffolds sequences larger than 200 bp and the open reading frame (ORF) was identified to obtain the corresponding protein sequence (58). Protein sequences were compared with those in the KEGG (http://www.genome.jp/kegg/) protein database using KAAS (KEGG Automatic Annotation Server, http://www.genome.jp/kaas-bin/kaas_main?prog=GHOSTX&way=s) for annotation and classification (select “for Prokayotes” in “GENES Data Set,” and default for other parameters). The generated annotation results were summarized and counted so as to obtain the annotation results and the corresponding abundance information of proteins corresponding to each metabolic pathway level. We define the fourth level of annotation, KO, as gene. Then, QIIME 1 (Quantitative Insights into Microbial Ecology, https://qiime.org) was used to analysis the functional genes relative abundance distribution table of each sample. Kaiju (https://github.com/bioinformatics-centre/kaiju) was used to compare the screened and filtered clean data set with protein sequences of bacteria in the NCBI-NR database (ftp://ftp.ncbi.nih.gov/blast/db/) to obtain the relative abundance of bacteria at each classification level (kingdom, phylum, class, order, family, genus, and species) (greedy5 approach with e-value < 0.001) (59). QIIME 1 was used to obtain the composition and abundance distribution table of all sample at each classification level.

### Alpha and beta diversity, and the influence of abiotic factors.

In our study, Shannon and Simpson indices were selected to represent the change of alpha diversity. In specific, Shannon index reflects the uncertainty of species prediction of randomly selected individuals in the community, and it is sensitive to richness. The Simpson index, which represents the probability that two randomly selected individuals in a community belong to the same species, is more influenced by evenness. These diversity indices were calculated using the “diversity” function of the “vegan” package (60). Taxonomic diversity was calculated using the species level abundance obtained from metagenomic sequencing and functional gene diversity was calculated using the KO group abundance annotated in the fourth level of the KEGG database. The correlation between alpha diversity and vanadium concentration was analyzed using Pearson’s correlation coefficient. The variation in community composition of taxa and functional genes among different treatments was illustrated by NMDS analysis based on Bray-Curtis distance (“metaMDS” function in “vegan” package), the significance of variation was illustrated by ANOSIM analysis (“anosim” function in “vegan” package). Mantel analysis was used for testing the correlation between taxonomic composition and functional genes composition (“mantel” function in “vegan” package). To eliminate the effect of housekeeping genes on functional redundancy, we removed the genes classified as “Cellular Processes,” “Genetic Information Processing” in KEGG database, and conducted NMDS analysis, ANOSIM analysis, and Mantel analysis. The contributions of earthworm intestinal content and soil physicochemical properties to community change were analyzed quantitatively by VPA with “varpart” function in “vegan” package (14).

### Nestedness estimation.

Two approaches were used to assess whether beta diversity among different samples was due to turnover or nestedness. Firstly, the “nestednodf” function in the “vegan” package was used to calculate the “nestedness measure based on overlap and decreasing fills” (NODF) value of communities under different taxonomic classification levels (phylum, class, order, family, and genus) in the worm gut and soil. The NODF value represents the degree to which taxa of a large community are contained within progressively smaller communities. Higher nestedness indicates that a less diverse community is a subset of a more diverse community. To explore the nestedness between earthworm intestinal and soil communities, and the nestedness between communities treated with different concentrations, all samples were manually grouped. One group emphasized the differences between communities of earthworms and soil habitats, and the other group emphasized the differences between communities of different vanadium concentrations in earthworm and soil. The NODF value calculated from the experimental data was compared with that from the null model. If the value of the experimental data was higher than that of the null model, this would indicate the communities that were nested (9). The null model was generated using the “commsim” function in the “vegan” package. In the second method, the “betapart” package was used to calculate the nestedness of taxa and functional gene composition among different samples in the worm gut and soil (61). We still divided these into two groups of different habitats and different concentrations. According to Carvalho et al. (8), beta diversity can be divided into nestedness and turnover and the sum of beta diversity caused by the two drivers respectively is total community beta diversity. Therefore, the “bray. Part” function of “betapart” was used to calculate the proportion of nestedness and turnover in different communities.

### Community assembly analysis.

The distribution pattern of community abundance can be used to reflect the assembly mechanism. To infer the community assembly process and analyses the contribution of neutral theory and niche theory to the community assembly of different samples, the taxa and gene rank abundance distribution of earthworm intestinal gut and soil were fitted by using the model consistent with neutral and niche theory respectively according to Guo et al. (62). Multiple models were used to enhance the credibility of the results. All models were evaluated by the AIC value that measures the relative quality of a statistical model. The model with the smallest AIC value is considered to be the optimal model. The ZSM model conforming to the neutral theory (63) was constructed by using Tetame (64). The Brokenstick, preemption, log-normal, and Zipf models are considered to represent niche theory (29) and were calculated using the “radfit” function in the “vegan” package (60). The AIC value was obtained using the formula AIC = −2 log-likehood + 2 × npar, where npar is the number of parameters in the fitting model (28). Through the calculation of the ZSM model, parameter *m* could be obtained and this represented the migration rate of species from a hypothetical species pool into a specific environment. The value of *m* was between 0 and 1. When *m *= 1, there is no dispersal limit, i.e., all species migrate from other places (65).

### Niche breadth.

We calculated niche breadth to reflect the adaptability and diversity pattern of the biological community (51). To reveal the distribution pattern of VDG-carrying taxa and VDGs in the earthworm intestines and soil, the Levins niche breadth index (*B*) (66) was calculated using the following formula:

$$B_{j}=1/\overset{N}{\underset{i=1}{\sum }}P_{ij}{}^{2}$$

Where *B_j_* represents the niche breadth of taxon *j* in the metacommunity, *N* refers to the total number of communities in the metacommunity, and *P_ij_* is the proportion of taxon *j* in community *i*. A higher value of *B* indicates taxa distributed evenly and widely, indicating a larger niche breadth. The niche breadth of a community is calculated as the average of *B* values from all taxa occurring in the community (52, 67). The above-mentioned calculation was carried out using the function of “niche. Width” in the package “spaa” (68).

### Data analysis.

All data analysis in this study were performed in R (v4.0.3; http://www.r-project.org/). ANOVA was used to determine the significant differences of parameters among samples. ANOSIM was performed to determine whether the difference showed by NMDS was significant. Significant differences of NODF values between experimental data and the null model were calculated using Wald test. TBtools (v1.082) (69) and the R package “ggplot2” (70) were used for depicting graphs.

### Data availability.

The metagenomic sequences supporting this work have been deposited in the Sequence Read Archive repository and can be accessed under BioProject PRJNA789344.
