# Supplementary material for: Metagenomic Sequencing Reveals that the Assembly of Functional Genes and Taxa Varied Highly and Lacked Redundancy in the Earthworm Gut Compared with Soil under Vanadium Stress
Source: mSystems. 2022 Jan 4;7(1):e01253-21. doi: 10.1128/mSystems.01253-21 (PMC8725585; doi:10.1128/mSystems.01253-21)
Supplement: TABLE S1 [file msystems.01253-21-st001.docx]

**Table S1** Mantel analysis for testing the correlation between taxonomic composition and functional genes composition in earthworm gut and soil.

|  | r | p |
| --- | --- | --- |
| Soil | 0.602 | 0.419 |
| Earthworm gut | 0.422 | 0.093 |

Notes: Significance analysis was based on 999 permutation tests. The Bray-Curtis distance was used to generate the distance matrix between samples. The statistic based on Spearman correlation coefficient.
