# Supplementary figures and images for: Metagenomic Sequencing Reveals that the Assembly of Functional Genes and Taxa Varied Highly and Lacked Redundancy in the Earthworm Gut Compared with Soil under Vanadium Stress
Source: mSystems. 2022 Jan 4;7(1):e01253-21. doi: 10.1128/mSystems.01253-21 (PMC8725585; doi:10.1128/mSystems.01253-21)

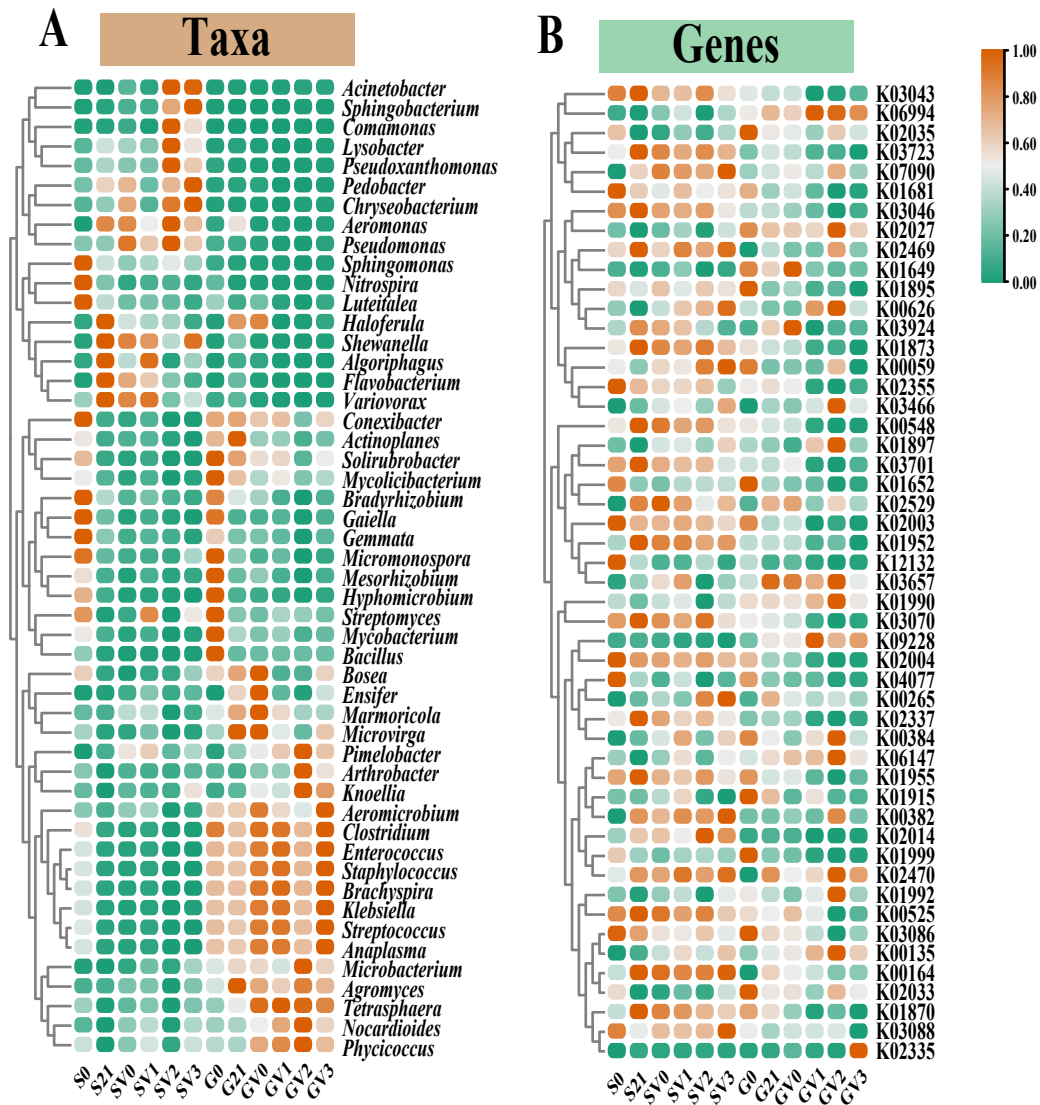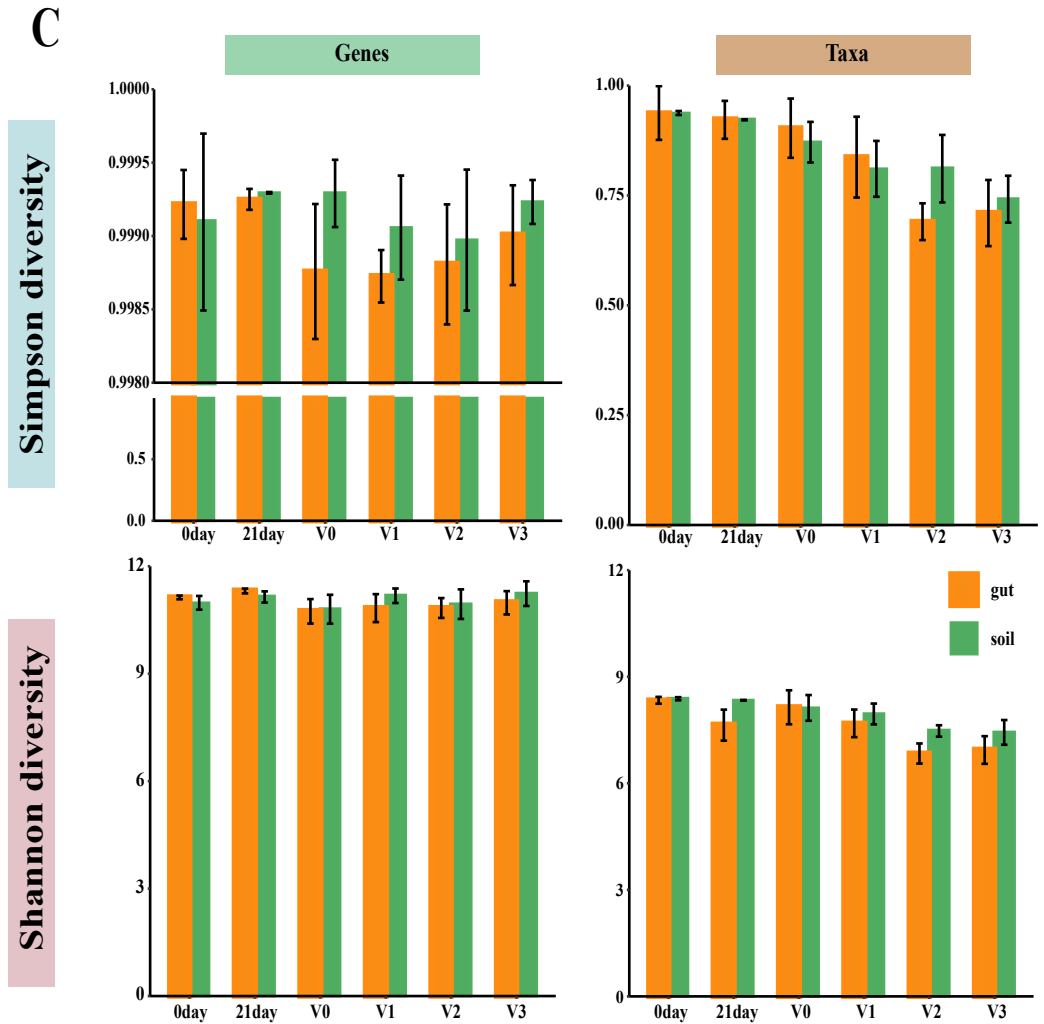

Supplement: FIG S2 [file msystems.01253-21-sf002.pdf]

A

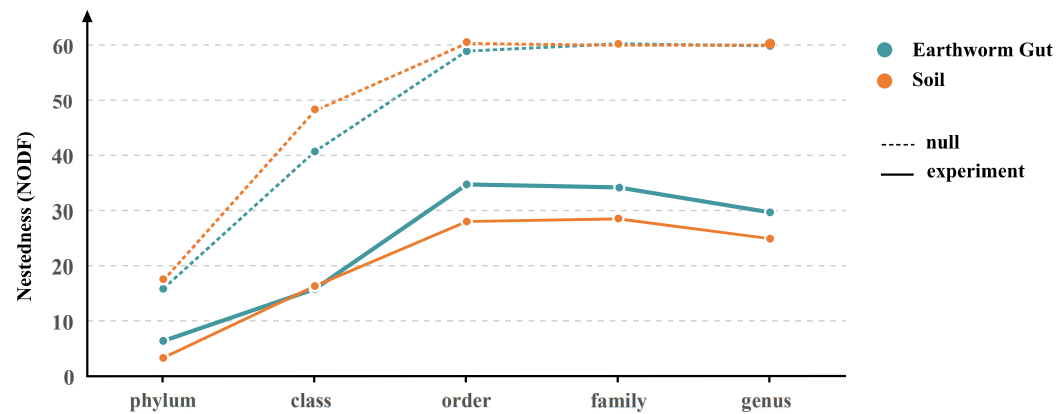

B

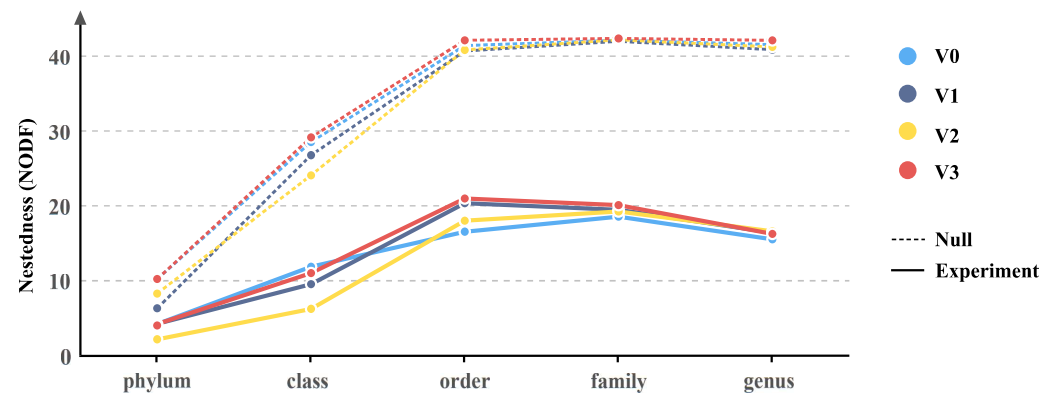

C

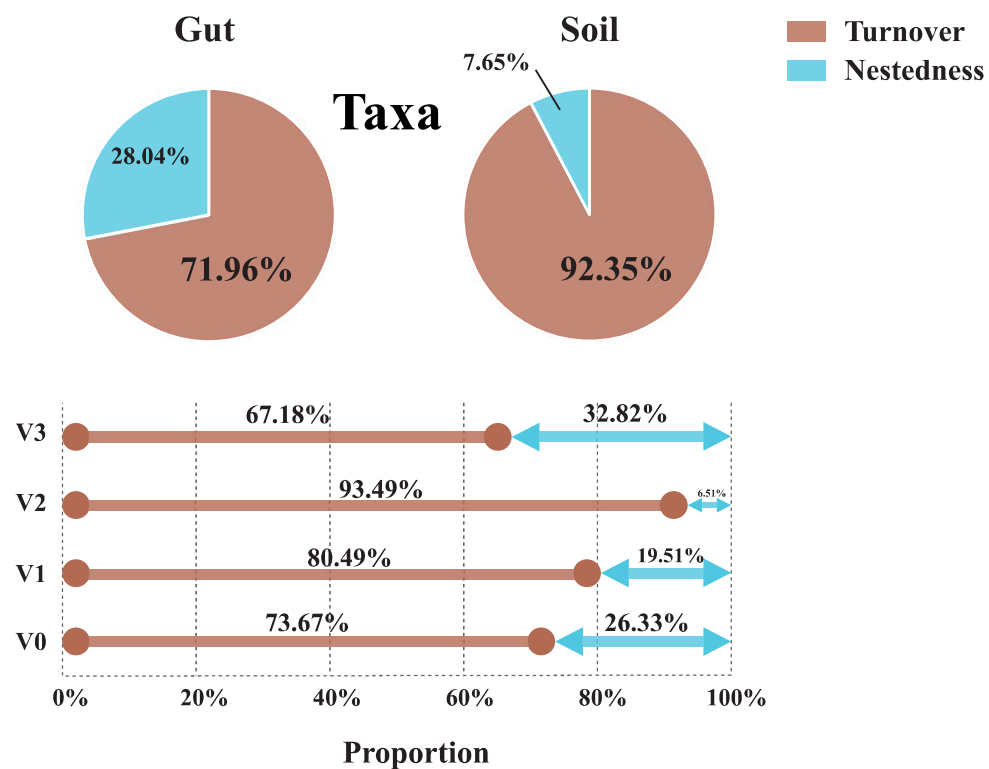

D

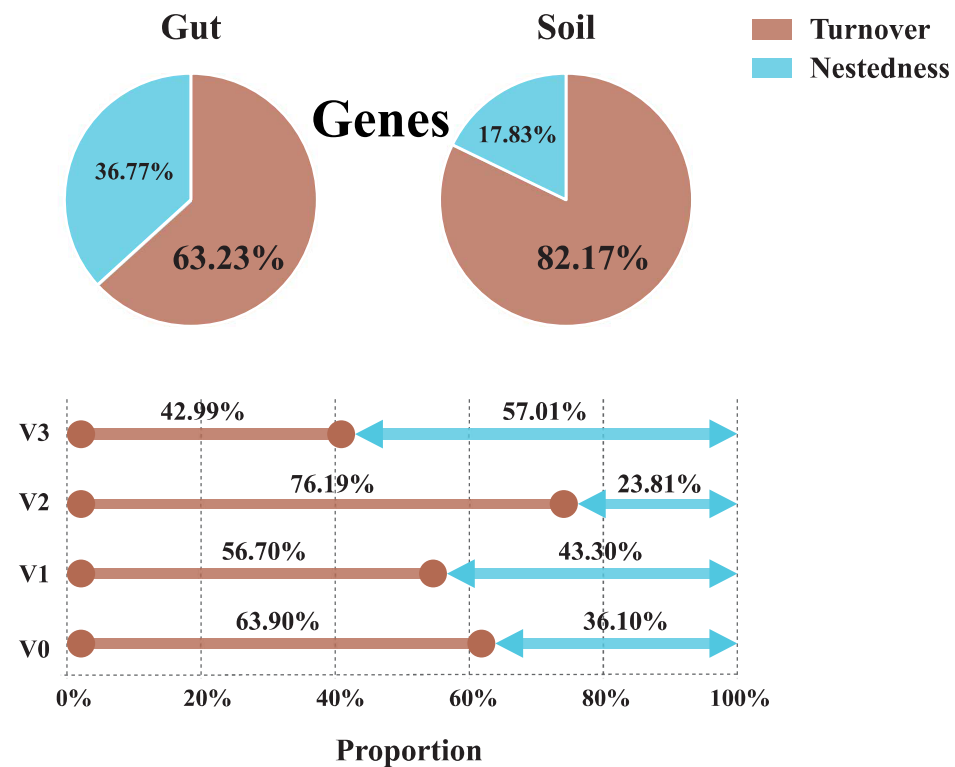

Supplement: FIG S3 [file msystems.01253-21-sf003.pdf]

A Gut

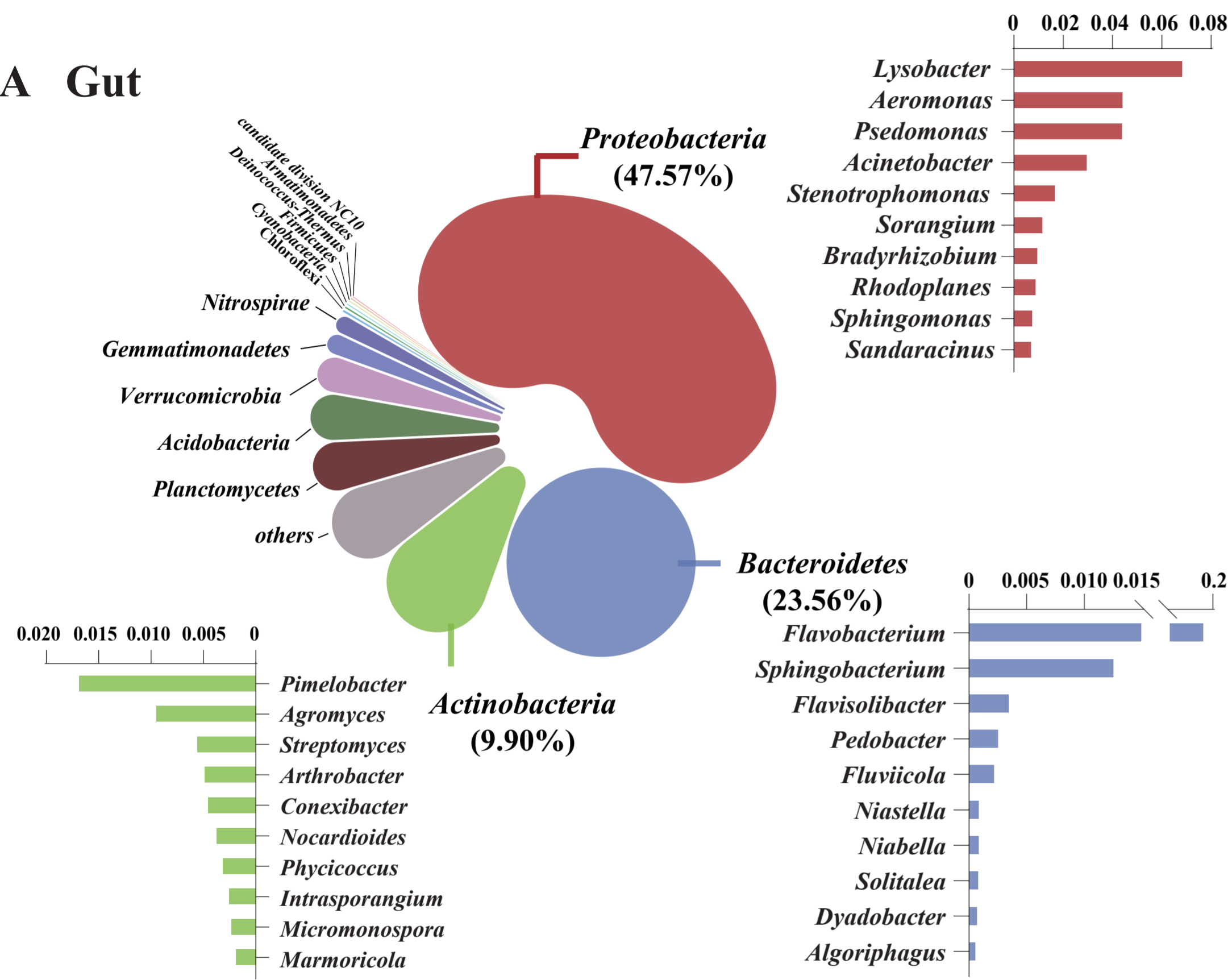

B Soil

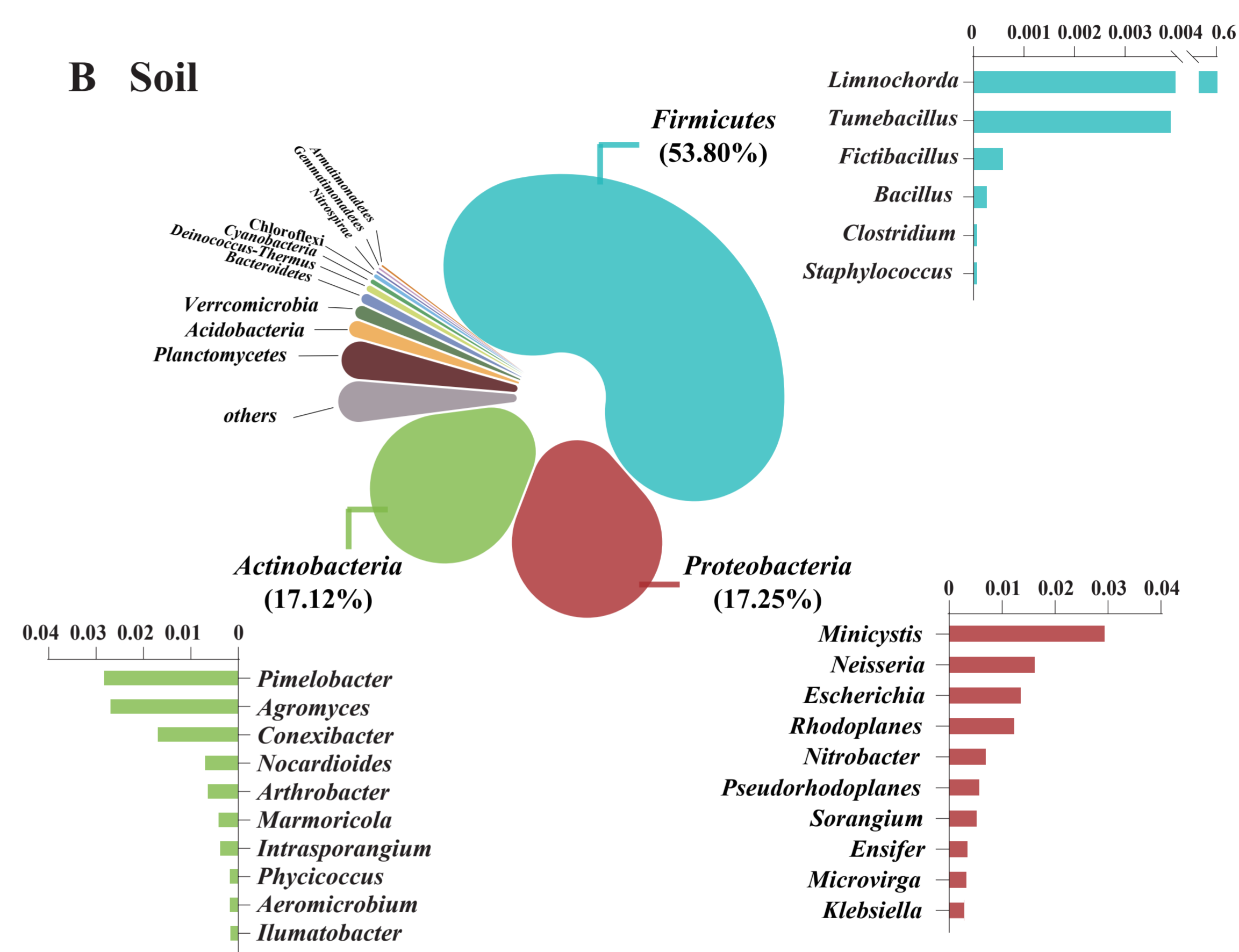

Supplement: FIG S4 [file msystems.01253-21-sf004.pdf]

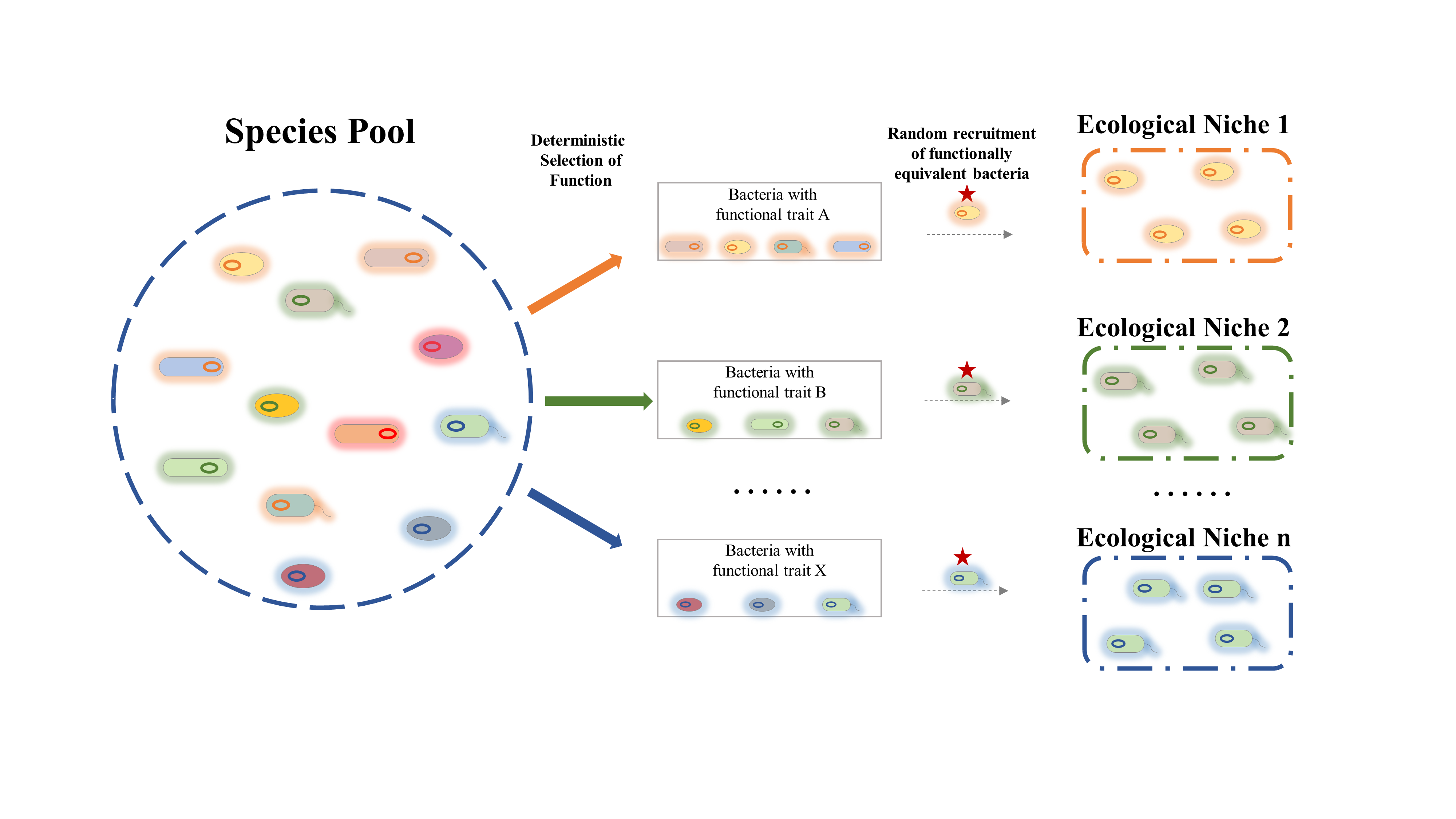

Supplement: FIG S5 [file msystems.01253-21-sf005.tif]

**A**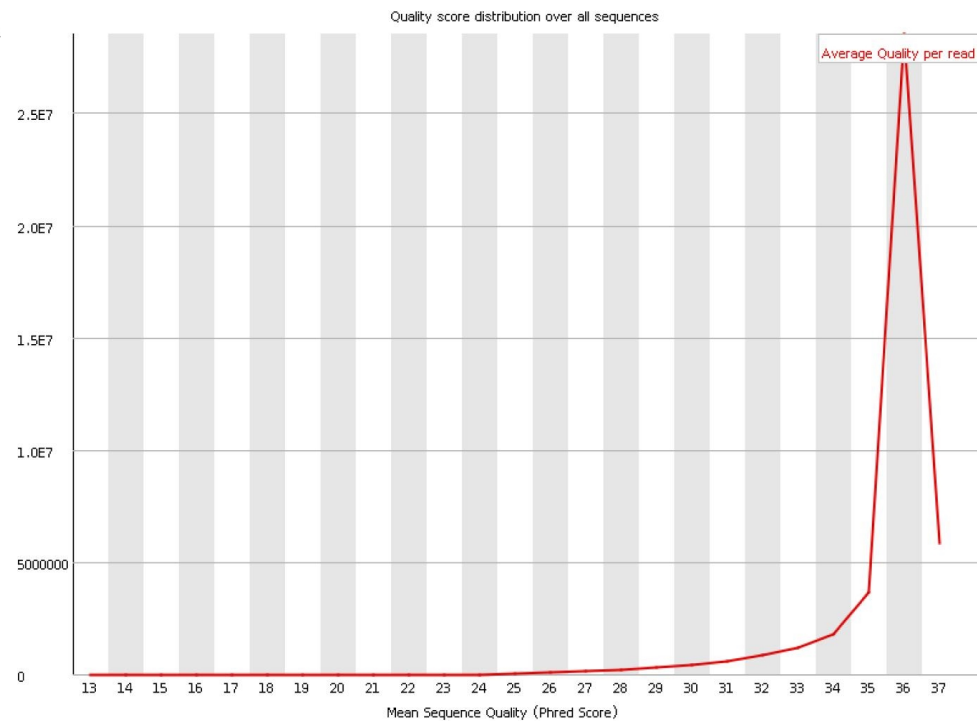**B**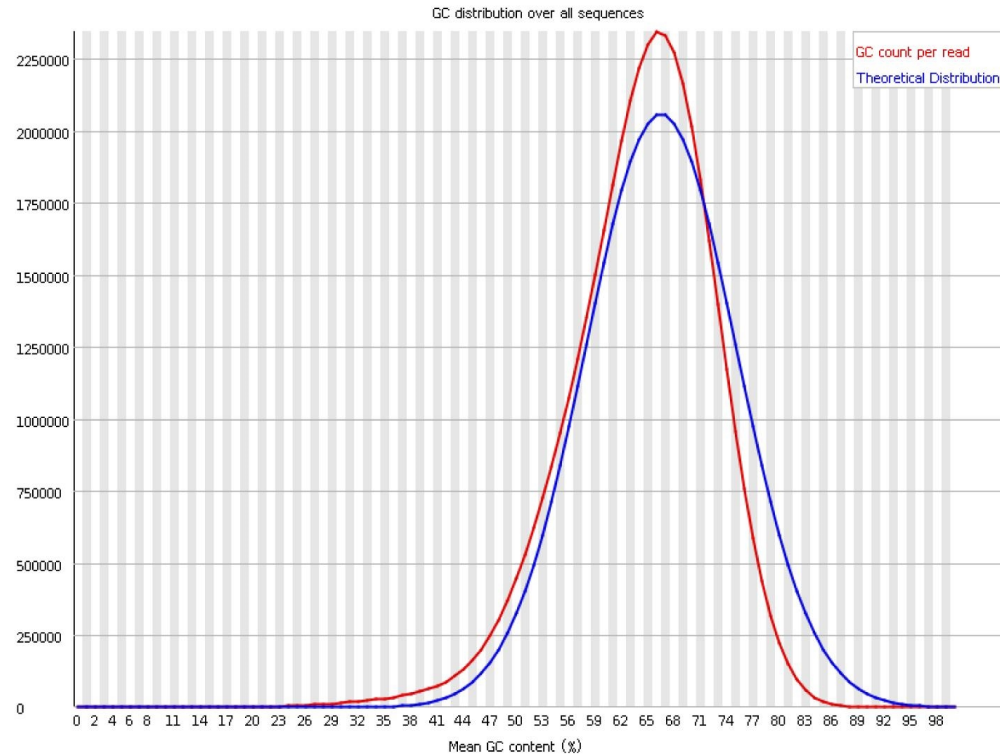**C**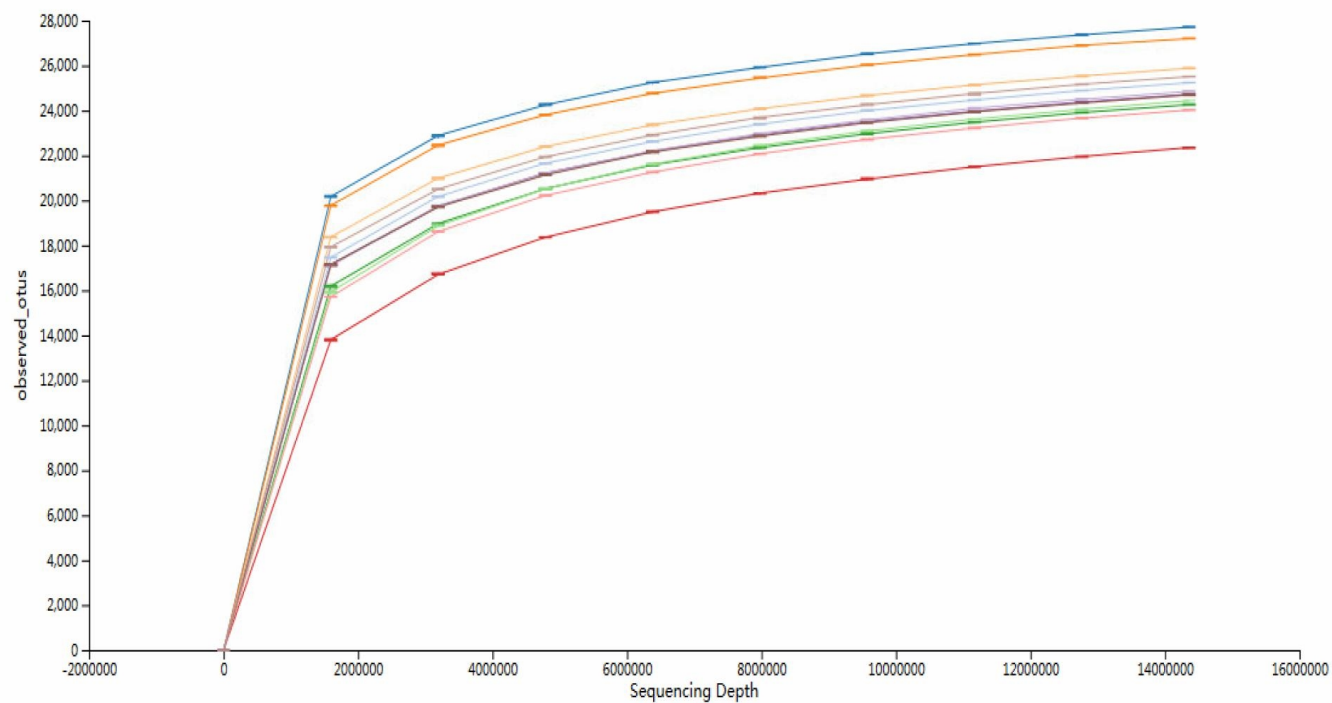

Supplement: FIG S1 [file msystems.01253-21-sf001.pdf]
