# Supplementary material for: Metagenomic Sequencing Reveals that the Assembly of Functional Genes and Taxa Varied Highly and Lacked Redundancy in the Earthworm Gut Compared with Soil under Vanadium Stress
Source: mSystems. 2022 Jan 4;7(1):e01253-21. doi: 10.1128/mSystems.01253-21 (PMC8725585; doi:10.1128/mSystems.01253-21)
Supplement: TABLE S2 [file msystems.01253-21-st002.docx]

**Table S2** ANOSIM analysis of the taxonomic/metabolic gene community composition between habitats (earthworm gut and soil) and vanadium concentration

|  | Factor | | Anosim | | |
| --- | --- | --- | --- | --- | --- |
|  |  |  | R | | *P* |
| Taxa | Habitat | | 0.753 | **< 0.001** | |
|  | vanadium concentration | earthworm gut | 0.037 | **< 0.001** | |
|  |  | soil | 0.125 | **0.007** | |
| Genes | Habitat | | 0.674 | **0.023** | |
|  | vanadium concentration | earthworm gut | 0.238 | **0.007** | |
|  |  | soil | 0.437 | 0.375 | |

Notes: Significance analysis was based on 999 permutation tests. Bold *P-*values indicate significant differences (*P* <0.05).
