# Supplementary material for: Metagenomic Sequencing Reveals that the Assembly of Functional Genes and Taxa Varied Highly and Lacked Redundancy in the Earthworm Gut Compared with Soil under Vanadium Stress
Source: mSystems. 2022 Jan 4;7(1):e01253-21. doi: 10.1128/mSystems.01253-21 (PMC8725585; doi:10.1128/mSystems.01253-21)
Supplement: TABLE S5 [file msystems.01253-21-st005.docx]

**Table S5** Five models used to determined the process of community assembly

| **Theory** | **Models** | **Description** | **Reference** |
| --- | --- | --- | --- |
| neutral theory | zero-sum multinomial (ZSM) | The model is consistent with two assumptions: first, the total number of individuals in the community is fixed, and the increase of the abundance of one species must be accompanied by the decrease of the same number of individuals of other species; The other is that individuals of all species have exactly the same rates of birth, death, dispersal and speciation. The expected species abundance in a local community of size *J* is:  $E_{local}\left\{ \left. r_{i} \right\vert J \right\}=\sum_{k=1}^{c} r_{i}\left( k \right)\cdot\varphi(k)$ | (McGill *et al.*, 2006) |
| niche theory | Brokenstick | The model assumes that the total ecological niche (total resources) in a community is a stick equal to 1. *N-1* points are randomly set on the stick, and the stick is divided into *N* segments, representing that the ecological niche is occupied by *N* species. In this model, it is assumed that *N* species have similar taxonomic status and similar competitive ability, and appear in the community at the same time. The total number of species in the community is *J*, then the abundance of the *i*th species in this model is:  $N_{i}=\frac{J}{N}\sum_{x=i}^{n} \frac{1}{x}$ | (Macarthur, 1957) |
|  | preemption | According to this model, the most dominant species in the community occupies *K* of the total ecological niche first, and the second dominant species occupies the remaining *K*, that is, *K (1 - K)*, and so on, until the remaining resources can no longer sustain the survival of a species. This model ensures that dominant species have priority in resource utilization and all species form a distinct hierarchy in niche occupation. *N_1_* represents the species abundance of the most dominant species in the model, then the abundance of the *i*th species in the model is:  ${N_{i}=N}_{I}{(1-k)}^{i-1}$ | (Whittaker, 1965) |
|  | log-normal | According to this model, the logarithm of the total number of individuals (*N*) in the community conforms to the normal distribution, so the abundance of the *i*th species is:  $N_{i}=e^{\left[ \log\left( \mu\right)+\log\left( \sigma\right)\emptyset\right]}$ | (Preston, 1948) |
|  | Zipf | This model holds that the cost of a species' existence depends on the current physical conditions and the species currently present. Pioneer species are low cost and require few prerequisites; Later species became scarcer because of the high cost of species, the demands on energy, time, and the structure of the ecosystem. This actually reflects a process of succession in which later settlers have a harder time surviving than those who arrived first. *P_1_* represents the proportion of the abundance of the richest species predicted by the model, γ is a constant, representing the average probability of species emergence, and the abundance *Ni* of the *i*th species can be expressed as:  $N_{i}=Jp_{1}i^{-\gamma}$ | (Frontier, 1985) |

**Reference:**

Frontier, S. (1985). DIVERSITY AND STRUCTURE IN AQUATIC ECOSYSTEMS. Oceanography and Marine Biology 23: 253-312.

Macarthur, R.H. (1957). ON THE RELATIVE ABUNDANCE OF BIRD SPECIES. Proc. Natl. Acad. Sci. U. S. A. 43: 293-295.

McGill, B.J., Maurer, B.A. and Weiser, M.D. (2006). Empirical evaluation of neutral theory. Ecology 87: 1411-1423.

Whittaker, R.H. (1965). Dominance and Diversity in Land Plant Communities: Numerical relations of species express the importance of competition in community function and evolution. Science (New York, N.Y.) 147: 250-260.
