# Supplementary material for: Metagenomic Sequencing Reveals that the Assembly of Functional Genes and Taxa Varied Highly and Lacked Redundancy in the Earthworm Gut Compared with Soil under Vanadium Stress
Source: mSystems. 2022 Jan 4;7(1):e01253-21. doi: 10.1128/mSystems.01253-21 (PMC8725585; doi:10.1128/mSystems.01253-21)
Supplement: TABLE S4 [file msystems.01253-21-st004.docx]

**Table S4** The KO numbers and corresponding names of vanadium detoxifying genes (VDGs) which falls into three different categories

| **Transport/efflux system** | | **Reductive metabolic pathway** | | **Oxidative damage repair** | |
| --- | --- | --- | --- | --- | --- |
| **KO number** | **Name** | **KO number** | **Name** | **KO number** | **Name** |
| K12952 | *ctpE+CC34:C50* | K00957 | *cysDP2: Q2P2: Q23* | K04564 | *SOD2* |
| K12953 | *ctpF* | K00860 | *cysC* | K04565 | *SOD1* |
| K12954 | *ctpG* | K00390 | *cysH* | K03781 | *katE* |
| K16264 | *czcD* | K00380 | *cysJ* | K00430 | *E1.11.1.7* |
| K15725 | *czcC* | K01738 | *cysK* | K00384 | *trxB* |
| K15726 | *czcA* | K00640 | *cysE* | K03671 | *trxA* |
| K15727 | *czcB* | K01758 | *CTH* | K03672 | *trxC* |
| K07787 | *cusA* | K00789 | *metK* | K03674 | *grxA* |
| K07796 | *cusC* | K17462 | *yrrT* | K03675 | *grxB* |
| K02013 | *ABC.FEV.A* | K01243 | *mtnN* | K03676 | *grxC* |
| K02014 | *TC.FEV.OM* | K07173 | *luxS* | K07390 | *grxD* |
| K02015 | *ABC.FEV.P* | K17216 | *mccA* | K07304 | *msrA* |
| K02016 | *ABC.FEV.S* | K00928 | *lysC* | K07305 | *msrB* |
| K02034 | *ABC.PE.P1* | K00133 | *asd* | K07147 | *msrP* |
| K02035 | *ABC.PE.S* | K12524 | *thrA* | K03673 | *dsbA* |
| K02074 | *ABC.ZM.A* | K00651 | *metA* | K03611 | *dsbB* |
| K02075 | *ABC.ZM.P* | K01739 | *metB* | K03981 | *dsbC* |
| K02077 | *ABC.ZM.S* | K01760 | *metC* | K04084 | *dsbD* |
| K09818 | *ABC.MN.S* | K00549 | *metE* | K03805 | *dsbG* |
| K09819 | *ABC.MN.P* | K01697 | *CBS* |  |  |
| K09820 | *ABC.MN.A* | K00383 | *GSR* |  |  |
| K15550 | *mdtP* | K00799 | *GST* |  |  |
| K18139 | *oprM* | K00681 | *GGT* |  |  |
| K18300 | *oprN* | K01255 | *CARP* |  |  |
| K18308 | *opmD* | K00370 | *narG* |  |  |
| K18903 | *oprC* | K00366 | *nirA* |  |  |
|  |  | K00368 | *nirK* |  |  |
|  |  | K04561 | *norB* |  |  |
|  |  | K00376 | *nosZ* |  |  |
|  |  | K02586 | *nifD* |  |  |
|  |  | K00266 | *gltD* |  |  |
|  |  | K01915 | *glnA* |  |  |
