# Supplementary material for: Metagenomic Sequencing Reveals that the Assembly of Functional Genes and Taxa Varied Highly and Lacked Redundancy in the Earthworm Gut Compared with Soil under Vanadium Stress
Source: mSystems. 2022 Jan 4;7(1):e01253-21. doi: 10.1128/mSystems.01253-21 (PMC8725585; doi:10.1128/mSystems.01253-21)
Supplement: TABLE S3 [file msystems.01253-21-st003.docx]

**Table S3**  Abiotic factors of earthworm intestinal contents and soil under different treatments

| Treatment | pH | Total Carbon  (g kg^-1^) | Total Nitrogen  (g kg^-1^) | Nitrate Nitrogen  (mg kg^-1^) | Ammonium Nitrogen  (mg kg^-1^) | Alkali-hydrolysable Nitrogen  (mg kg^-1^) | Available Phosphorus  (mg kg^-1^) | Available Sulfur  (mg kg^-1^) |
| --- | --- | --- | --- | --- | --- | --- | --- | --- |
| G0 | 7.1±0.4a | 95.2±4.7c | 8.8±0.4ab | 15.8±3.3b | 0.3±0.03c | 62.5±1.4b | 12.8±3.7a | 19.6±5.6a |
| G21 | 7.0±0.07a | 105.0±1.9b | 9.4±1.1a | 18.4±4.5ab | 0.7±0.05b | 70.3±5.6a | 9.2±2.6a | 20.3±4.5a |
| GV0 | 6.7±0.06a | 108.4±4.3ab | 8.4±2.4b | 20.3±1.3a | 0.9±0.07ab | 73.3±4.6a | 10.3±1.6a | 23.5±3.5a |
| GV1 | 7.0±0.02a | 113.5±2.0a | 9.3±1.7a | 18.7±1.5ab | 1.0±0.01a | 72.2±7.7a | 11.4±2.7a | 22.4±6.7a |
| GV2 | 7.3±0.02a | 110.0±1.7ab | 8.7±1.2ab | 16.2±0.6ab | 1.0±0.02ab | 69.8±6.7a | 12.7±2.9a | 21.9±5.6a |
| GV3 | 7.3±0.04a | 105.7±3.2ab | 8.2±2.5c | 17.6±2.1b | 0.8±0.10ab | 75.7±3.9a | 11.5±3.8a | 22.7±4.6a |
| S0 | 6.7±0.02a | 63.8±3.4b | 1.7±1.2a | 5.3±2.1a | 2.5±1.6b | 65.4±4.8b | 12.1±2.1a | 15.9±3.6a |
| S21 | 6.7±0.03a | 71.3±5.9ab | 1.9±1.0a | 6.2±3.4a | 8.3±2.5a | 68.7±3.7ab | 15.6±2.3a | 17.8±2.9a |
| SV0 | 6.6±0.03a | 74.7±4.3ab | 1.7±0.7a | 6.7±1.9a | 9.2±2.5a | 71.9±6.3a | 16.2±4.2a | 17.6±2.3a |
| SV1 | 6.7±0.04a | 81.5±3.3a | 1.8±1.1a | 5.8±2.6a | 10.1±1.6a | 73.5±4.3a | 16.4±2.2a | 16.8±3.4a |
| SV2 | 6.7±0.02a | 76.4±2.6a | 1.7±1.3a | 5.0±1.4a | 10.8±1.2a | 72.4±7.7a | 15.9±1.5a | 17.9±3.0a |
| SV3 | 6.7±0.03a | 73.9±5.7ab | 1.6±1.2a | 4.7±1.7a | 8.6±3.2a | 62.8±6.5b | 13.1±2.4a | 16.3±2.1a |

Note: G0, the original worm gut content without microcosm trial; G21, the worm gut content sampled at day 21 of the incubation; the earthworm gut contents treated with 0, 100, 200 and 300 mg kg^-1^ vanadium after 21days of incubation are represented by GV0, GV1, GV2, GV3, respectively. S0, the original soil without microcosm trial; S21, the soil sampled at day 21 of the incubation; the soils treated with 0, 100, 200 and 300 mg kg^-1^ vanadium after 21days of incubation are represented by SV0, SV1, SV2, SV3, respectively. Values within the same column followed by uniform letters indicate no significant difference (P > 0.05, ANOVA, Tukey’s HSD test).
